# Quercetin, a flavonoid, suppresses viral proliferation by interfering with the ubiquitin transfer from E1 to E2 enzymes

**DOI:** 10.1371/journal.ppat.1014425

**Published:** 2026-07-20

**Authors:** Hanbo Li, Yu Li, Bowen Yin, Xinming Zhang, Zheng Xu, Changxu Song, Kang Li, Ling Tian

**Affiliations:** 1 Guangdong Provincial Key Laboratory of Agro-animal Genomics and Molecular Breeding, College of Animal Science, South China Agricultural University, Guangzhou, China; 2 Guangdong Engineering Technology Research Center of Sericulture, College of Animal Science, South China Agricultural University, Guangzhou, China; 3 School of Life Science and Technology, China Pharmaceutical University, Nanjing, China; 4 National Engineering Center for Swine Breeding Industry, South China Agricultural University, Guangzhou, China; 5 School of Agriculture, Guangxi University, Nanning, China; 6 Guangxi Key Laboratory of Sericulture Ecology and Applied Intelligent Technology/Guangxi Collaborative Innovation Center of Modern Sericulture and Silk, Hechi University, Hechi, China; KU: The University of Kansas, UNITED STATES OF AMERICA

## Abstract

Quercetin is recognized for diverse pharmacological activities. However, the mechanism underlying its broad-antiviral effects has not been elucidated. Herein, we identified quercetin as a potent inhibitor of both double-stranded DNA virus *Bombyx mori* nucleopolyhedrovirus (BmNPV) and single-stranded RNA virus porcine reproductive and respiratory syndrome virus (PRRSV). Surface plasmon resonance (SPR) revealed that quercetin targets host ubiquitin-activating enzyme 1 (Uba1) homologs. *Uba1* knockdown reduced viral proliferation and enhanced the antiviral effect of quercetin, whereas *Uba1* overexpression functioned oppositely. Quercetin bound Uba1 homologs with high affinity. Notably, mutation of two binding residues, Q977 and G978, significantly disrupted the binding between BmUba1 and quercetin, and abolished quercetin’s antiviral activity. Quercetin obstructed the transfer of ubiquitin from Uba1 to the E2 enzyme Ubc6, impairing the ubiquitination process. Similarly, quercetin inhibited PRRSV proliferation via targeting Uba1 in mammals. These findings elucidate the molecular mechanism underlying the pharmacological effects of quercetin, providing a theoretical basis for the development of novel antiviral agents against both DNA and RNA viruses.

## Author summary

Focusing on BmNPV and PRRSV, this study reveals that quercetin inhibits both DNA and RNA viruses by targeting Uba1 homologs, disrupting ubiquitin transfer between E1 and E2 enzymes. Mutations in key residues on Uba1 impair the binding with quercetin, thus lost the antiviral effect of quercetin. The conserved mechanism across species underscores quercetin’s broad-spectrum antiviral potential, providing a theoretical basis for developing effective antiviral drugs against diverse viral infections.

## Introduction

Quercetin, a naturally derived flavonoid abundant in plants, exhibits diverse and potent biological activities, making it a promising candidate for therapeutic applications in cardiovascular diseases, cancer, metabolic disorders, and infectious diseases [1,2]. Its broad-spectrum antiviral properties are of particular interest [3]. For instance, quercetin inhibits the synthesis and secretion of hepatitis B virus (HBV) surface and e antigens to suppress the viral proliferation in mammals [4,5]. Additionally, it demonstrates antioxidant activity by reducing the production of reactive oxygen and nitrogen species (ROS/RNS) and counteracting lipid peroxidation induced by hepatitis C virus (HCV) infection in human cells [6]. Quercetin exerts its antiviral effect against Porcine Reproductive and Respiratory Syndrome Virus (PRRSV) by inhibiting the formation of the viral replication and transcription complex (RTC) and downregulating the expression of *heat shock protein 70* (*Hsp70*), consequently suppressing viral replication [4]. Moreover, quercetin modulates inflammatory responses in cells infected by herpes simplex virus (HSV) by specifically decreasing *toll-like receptor 3* (*TLR-3*) expression, which subsequently inhibits the activation of key inflammation-related factors such as interferon regulatory factor 3 (IRF3) and nuclear factor kappa-B (NF-κB) [7]. Quercetin exhibits potent broad-spectrum antiviral activity against viruses from invertebrates to mammals, yet the underlying molecular mechanisms remain poorly defined.

Ubiquitination plays a pivotal role in numerous cellular processes, including transcription, cell cycle, stress responses, DNA repair, apoptosis, immune responses, and autophagy [8–10]. Ubiquitination is an ATP-dependent biochemical process wherein E1 enzymes activate ubiquitin, transfer it to E2 enzymes, and E3 ligases facilitate the final attachment of ubiquitin to target proteins [11]. It also serves a critical function in the virus-host interaction. For instance, the human immunodeficiency virus (HIV) protein Vif (viral infectivity factor) exploits ubiquitin modification to degrade the host restriction factor APOBEC3G (apolipoprotein B mRNA editing enzyme catalytic polypeptide-like 3G), thereby promoting viral replication [12]. Conversely, the host E3 ubiquitin ligase TRIM5α specifically recognizes and subsequently ubiquitinates the HIV capsid protein to suppress infection [13]. Multiple small molecules targeting the ubiquitin activation system have been reported to exert inhibitory effects on pathogens in experimental studies. For example, the small molecule LS-102 serves as an inhibitor of dengue virus infection by blocking the interaction between viral non-structural protein 4A (NS4A) and the E3 enzyme hydroxymethylglutaryl reductase degradation protein 1 (HRD1), while the microbial metabolite APL-16–5 binds to the E3 enzyme TRIM25, promoting ubiquitination and degradation of the influenza A virus (IAV) polymerase subunit PA [14,15]. Whereas, the precise mechanism by which the ubiquitin-proteasome system mediates virus-host interactions remains largely elusive.

Baculoviruses, characterized by circular double-stranded DNA, primarily infect insect hosts via a foodborne route and exhibit substantial potential for pest biological control and the production of mammalian adeno-associated viruses (AAV) and recombinant proteins [16–19]. *Bombyx mori* nucleopolyhedrovirus (BmNPV) is a major pathogen in sericulture, accounting for approximately 60% of diseases in domestic silkworms and causing substantial economic losses [20,21]. Silkworms deploy multiple defense pathways, such as RNA interference, NF-κB-mediated signaling, and the JAK/STAT pathway, to resist BmNPV proliferation [22]. In comparison, BmNPV manipulates host signaling pathways to facilitate its replication, including activating the PI3K/Akt and ERK pathways, inhibiting prophenol oxidase (PPO) activity, and promoting cytoplasmic-nucleo translocation of the transcription factor EB (BmTFEB) [22,23]. Although anti-BmNPV silkworm varieties have been developed through selective breeding and hybridization [24,25], there is no effective pharmacological intervention available to cure the infected larvae even among these anti-BmNPV silkworm varieties currently. PRRSV, a positive - sense single-stranded RNA virus, is a major pathogen in the swine industry, specifically targeting host macrophages and causing respiratory disease and immune dysfunction [26,27]. Currently, vaccines serve as the primary means of preventing PRRSV infection, but the inherently high mutation and recombination rates of RNA viruses result in limited protection duration and insufficient cross-strain protection [28,29]. Therefore, it is necessary to develop novel antiviral strategies to alleviate the economic and health issues caused by PRRSV.

Herein, quercetin was identified as a potent inhibitor of both BmNPV and PRRSV proliferation. Using these drug-host-virus interaction models, the ubiquitin-proteasome system was identified as the main pathway mediating the antiviral activity of quercetin against DNA and RNA viruses, conserved across insects to mammals. The findings shed light on the therapeutic strategy for combating infectious viruses, and provided effective prevention and treatment strategies for related diseases.

## Results

### Quercetin suppresses the proliferation of DNA virus BmNPV in silkworms

**Fig 1:**
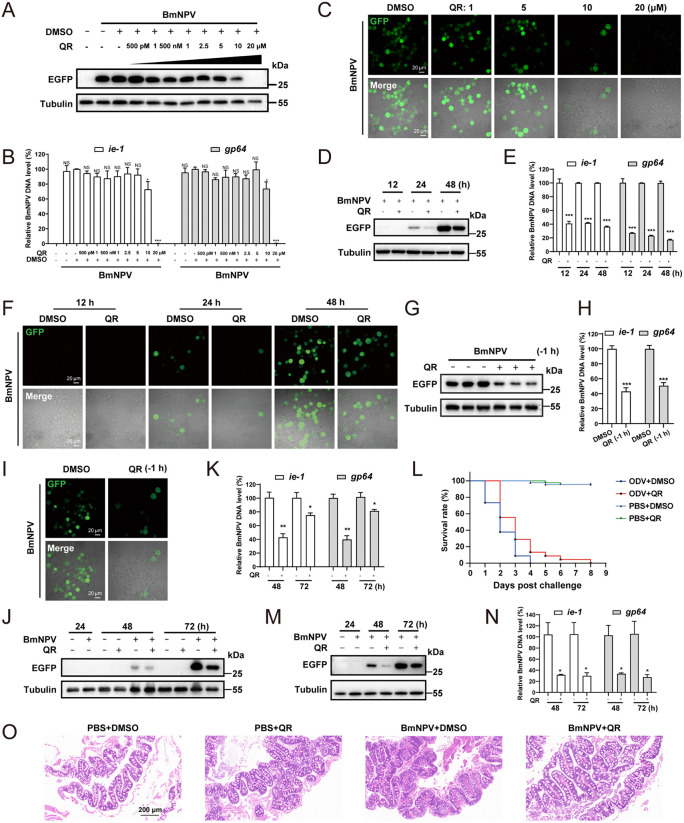
Inhibitory effect of quercetin (QR) on the proliferation of BmNPV. **(A-C)** After a 12-h infection with recombinant BmNPV-EGFP, BmN cells were treated with dimethyl sulfoxide (DMSO) or gradient doses of QR for further 36 h. Western blot analysis of EGFP protein **(A)**. Detection of BmNPV *ie-1* and *gp64* gene copies by qPCR **(B)**. Fluorescence observation of EGFP protein under a confocal microscope **(C)**. **(D-F)** After a 12-h infection with BmNPV-EGFP, BmN cells were treated with DMSO or 10 μM QR, and the viral load was subsequently assessed at 12 h, 24 h, and 48 h post QR treatment. Western blot analysis of EGFP protein **(D)**. Detection of BmNPV *ie-1* and *gp64* gene copies by qPCR **(E)**. Fluorescence observation of EGFP protein **(F)**. (G-I) BmN cells were pre-incubated with 10 μM QR for 1 h before BmNPV infection (-1 h). Western blot analysis of EGFP protein **(G)**. qPCR analysis of BmNPV *ie-1* and *gp64* **(H)**. Fluorescence observation of EGFP protein **(I)**. **(J, K)** After a 24-h infection with BmNPV, the silkworms were injected with QR (6 μg/ larva), and the viral load was subsequently evaluated at 24 h, 48 h, or 72 h post the viral infection. Western blot analysis of EGFP protein in the fat body **(J)**. qPCR analysis of BmNPV *ie-1* and *gp64* in the fat body **(K)**. **(L)** Survival rate of silkworm larvae assessed across four experimental groups: (1) oral infection with ODV (5.46 × 10^9^/larva), followed by daily oral administration of DMSO (ODV+DMSO); (2) feeding with PBS, followed by daily oral administration of DMSO (PBS+DMSO); (3) oral infection with ODV, followed by daily oral administration of 100 μg QR per larva (ODV + QR); and (4) feeding with PBS, followed by daily oral administration of QR (PBS + QR). The treatment with QR or DMSO was conducted daily starting from day 1 post infection until the larvae died. A log-rank (Mantel-Cox) test was performed between ODV+DMSO and ODV + QR groups, revealing a statistically significant difference (*p* = 0.0007). **(M, N)** Following the same treatment as in Fig 1J and 1K, western blot analysis of EGFP protein in the midgut **(M)**. qPCR analysis of BmNPV *ie-1* and *gp64* in the midgut **(N)**. **(O)** After a 12-h infection with ODV, the midgut of silkworms, treated with 100 μg QR per larva or DMSO for 72 h, was collected and subsequently observed under a microscopy after undergoing hematoxylin-eosin (HE) staining. Data are presented as mean ± standard error (SE) (n = 3). Significant differences were evaluated by unpaired Student’s *t*-test **(B, E, H, K and N)**. *, *p* < 0.05; **, *p* < 0.01; ***, *p* < 0.001; NS, no significance.

Through screening a small-molecule compound library in our laboratory, we identified that quercetin can effectively inhibit the proliferation of BmNPV (S1 Data). Western blot analysis of EGFP protein levels in BmN cells post infection with recombinant BmNPV-EGFP revealed that quercetin exhibited antiviral effects at the micromolar range across a concentration gradient of 500 pM to 20 μM. Specifically, treatment with 10 μM quercetin significantly reduced EGFP protein levels, while at 20 μM, the protein was nearly undetectable compared to the control group (Figs 1A and S1A). Consistently, qPCR results showed that the gene copies of BmNPV *ie-1* and *gp64* were significantly decreased following treatment with 10 μM and 20 μM quercetin (Fig 1B). Accordingly, observation of BmNPV-EGFP fluorescence revealed that virus proliferation was significantly suppressed after treatment with 10 μM or 20 μM quercetin in BmN cells (Figs 1C and S1B). The cell survival assay showed that quercetin displayed relatively low cytotoxicity, with a 50% cytotoxic concentration (CC_50_) of 69.72 μM in BmN cells. Meanwhile, the selectivity index (SI) of quercetin was 9.47 (CC_50_ = 69.72 μM/ IC_50_ = 7.36 μM). These findings indicate that quercetin can effectively combat viruses without causing significant harm to host cells, thereby maintaining a favorable safety profile (S1C and S1D Fig). Therefore, we further detected the effect of 10 μM quercetin on virus proliferation at 12 h, 24 h, and 48 h after BmNPV infection. The results showed that the drug significantly inhibited the proliferation and replication of BmNPV, as evidenced by a notable reduction of EGFP protein levels, diminished EGFP fluorescence, and decreased gene copies of the viral *ie-1* and *gp64* (Figs 1D-1F, S1F and S1G). Similarly, pretreating with 10 μM quercetin for 1 h also reduced the proliferation of BmNPV-EGFP in BmN cells (Figs 1G-1I, S1H and S1I). Taken together, these findings suggested that quercetin effectively prevents BmNPV replication.

Consequently, the antiviral activity of quercetin in *B. mori* larvae was further investigated. Given that the fat body is the preferred tissue for BmNPV proliferation *in vivo* [30], we detected the viral load in this tissue. After injecting BmNPV-EGFP for 24 h, the larvae were injected with quercetin (6 μg/ larva) for an additional 24 h or 48 h. The results showed that quercetin treatment significantly reduced the replication and proliferation of BmNPV in the fat body (Figs 1J and 1K, S1J). After being orally infected with a lethal dose of occlusion-derived virus (ODV) for 24 h, the larvae were orally treated with quercetin at a dosage of 100 μg per larva. Remarkably, treatment with quercetin extended the survival period of silkworms following BmNPV infection, with certain larvae persisting until the eighth day post infection and successfully progressing through the spinning period. In contrast, all larvae in the control group succumbed on the fourth day after the viral infection. A log-rank (Mantel-Cox) test was performed between ODV+DMSO and ODV + Quercetin groups, revealing a statistically significant difference (*p* = 0.0007). (Fig 1L). Midgut serves as the primary physical barrier against orally ingested baculovirus, which subsequently spread to other tissues following the infection of midgut epithelial cells [31]. Therefore, we further analyzed the antiviral effect of quercetin in silkworm midgut tissues. The results showed that quercetin significantly reduces viral protein levels and DNA copies in a time-dependent manner (Figs 1M and 1N, S1K). Meanwhile, microscopic observation revealed that after ODV infection for 72 h, the boundaries of midgut tissue in silkworm larvae became indistinct, cell arrangement was disrupted, and the vacuoles within the goblet cells underwent shrinkage, with a reduction in both size and number. In contrast, quercetin treatment significantly alleviated these abnormalities in midgut tissue (Fig 1O). Taken together, quercetin effectively suppress BmNPV proliferation both *in vitro* and *in vivo* in silkworms.

### Screening for active targets of quercetin in *B. mori* through surface plasmon resonance analysis

**Fig 2:**
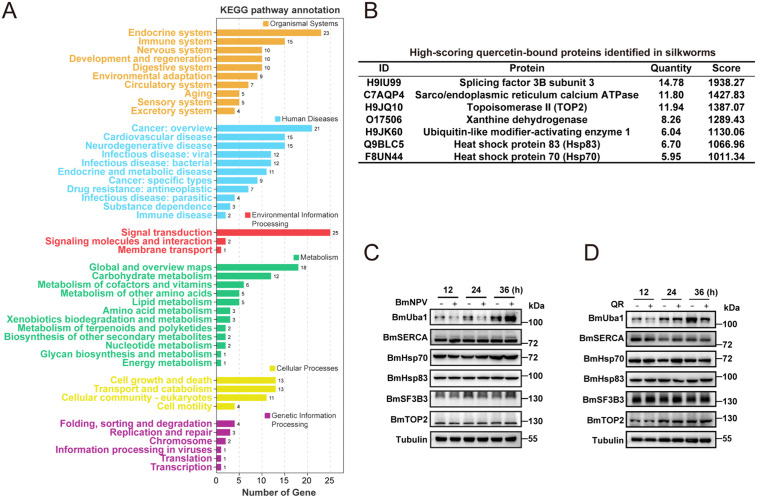
Screening for active targets of QR in BmN cells by SPR. **(A)** KEGG pathway annotation of proteins captured by QR molecule. **(B)** List of top-scoring candidate proteins and their relative quantity. **(C, D)** Western blot analysis of the six candidate proteins captured by QR in BmN cells after BmNPV-EGFP infection (C) or 10 μM QR treatment (D) at the specified time periods.

To ascertain the active targets of quercetin in its antiviral activity against BmNPV, SPR coupled with HPLC-MS/MS was employed to screen quercetin-binding proteins in *B. mori*. The binding interaction between quercetin and the lysates from BmN cells infected with BmNPV for 48 h was evaluated using SPR, and 101 proteins were identified through mass spectrometry (MS) (S2 Data). Kyoto Encyclopedia of Genes and Genomes (KEGG) pathway annotation revealed that the quercetin-binding proteins were involved in multiple key biological processes, mainly including endocrine system, immune system, cancer, cardiovascular disease, and signal transduction (Fig 2A), all of which were closely associated with the diverse pharmacological activities of quercetin in disease treatment [32]. Notably, the top 20 enriched pathways were implicated in various diseases, such as viral infection, cardiovascular diseases, metabolic disorders, and cancer (S2A Fig). Based on the score ranks and relative abundances, six candidate proteins with scores greater than 1,000, including splicing factor 3B subunit 3 (SF3B3), sarco/endoplasmic reticulum calcium ATPase (SERCA), topoisomerase II (TOP2), Uba1, heat shock protein 83 (Hsp83), and Hsp70, were selected as potential targets for further research (Figs 2B and S2B). Subsequently, we detected the protein levels of the six aforementioned targets in BmN cells after BmNPV infection. Results showed that only BmUba1, among the six proteins examined, was significantly upregulated at 36 h post infection, indicating a potential role of BmUba1 in the cellular response to BmNPV infection (Figs 2C and S2C). In comparison, quercetin treatment resulted in a significant reduction in BmUba1 protein levels at 36 h (Figs 2D and S2D). Meanwhile, the specificity of the anti-Uba1 antibody was verified via *BmUba1* knockdown and overexpression assays (S2E and S3B Fig).

### BmUba1 plays a regulatory role in BmNPV proliferation and mediates the antiviral activity of quercetin

**Fig 3:**
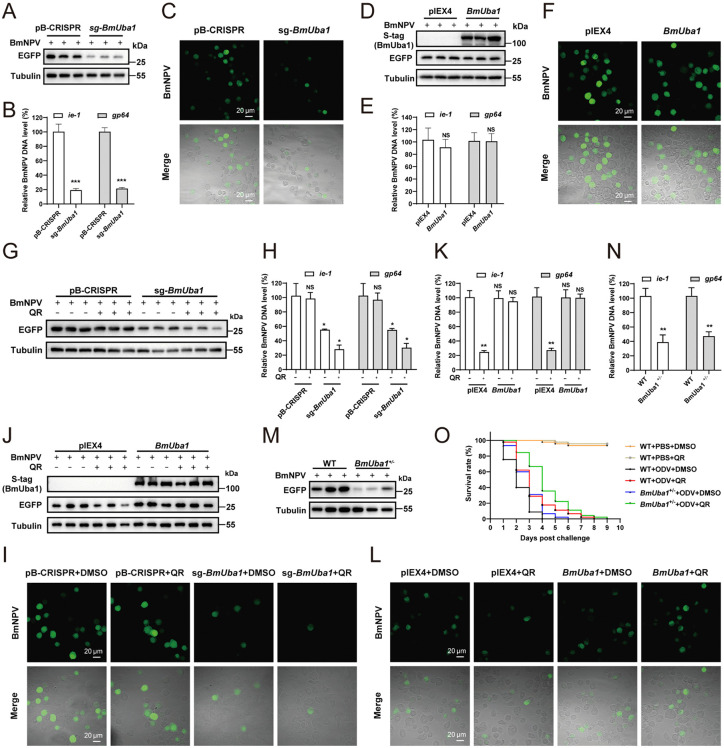
The impact of BmUba1 on BmNPV replication and the antiviral efficacy of QR. **(A-C)** Detection of BmNPV proliferation at 36 h post its infection in the *BmUba1*-knockdown BmN cells. Western blot analysis of EGFP protein **(A)**. qPCR analysis of BmNPV *ie-1* and *gp64* **(B)**. Fluorescence observation of GFP protein **(C)**. **(D-F)** Detection of BmNPV proliferation at 36 h post the viral infection in the *BmUba1* overexpressing BmN cells. Western blot analysis of EGFP protein **(D)**. qPCR analysis of BmNPV *ie-1* and *gp64* **(E)**. Fluorescence observation of GFP protein **(F)**. (G-I) After a 12-h infection with BmNPV, the *BmUba1*-knockdown cells were treated with DMSO or 5 μM QR for 36 **h.** Subsequently, the cells were collected for detecting the viral proliferation. Western blot analysis of EGFP protein **(G)**. qPCR analysis of BmNPV *ie-1* and *gp64* **(H)**. Fluorescence observation of GFP protein **(I)**. **(J-L)** After a 12-h infection with BmNPV, the *BmUba1*overexpressing BmN cells were treated with DMSO or 10 μM QR for 36 **h.** Subsequently, the cells were collected for detecting the viral proliferation. Western blot analysis of EGFP protein **(J)**. qPCR analysis of BmNPV *ie-1* and *gp64* **(K)**. Fluorescence observation of GFP protein **(L)**. **(M, N)** After a 48-h infection, the proliferation of BmNPV in silkworms with wild-type (WT) *BmUba1* or heterozygous-mutated *BmUba1* (*BmUba1*^*+/-*^) genotype were detected. Western blot analysis of EGFP protein in fat body by **(M)**. qPCR analysis of BmNPV *ie-1* and *gp64* in fat body **(N)**. **(O)** Survival rate of silkworm larvae with WT or *BmUba1*^*+/-*^ genotype was assessed across four experimental groups: WT- or *BmUba1*^*+/-*^-genotype larvae infected with ODV followed by 100 μg QR oral administration per larva (WT/*BmUba1*^*+/-*^ + ODV + QR) or DMSO (WT*/BmUba1*^*+/-*^ + ODV+DMSO) daily starting from day 1 post infection until the larvae died. Log-rank (Mantel-Cox) tests were conducted between WT + ODV+DMSO and *BmUba1*^*+/-*^ + ODV+DMSO groups (*p* = 0.0022), as well as between *BmUba1*^*+/-*^ + ODV+DMSO and *BmUba1*^*+/-*^ + ODV + QR groups (*p* = 0.0001), revealing statistically significant difference. Data are presented as mean ± SE (n = 3). Significant differences were evaluated by unpaired Student’s *t*-test **(B, E, H, K, N)**. *, *p* < 0.05; **, *p* < 0.01; ***, *p* < 0.001; NS, no significance.

To delve into the roles of the target BmUba1 in the antiviral activity of quercetin, we first examined the effect of *BmUba1* knockdown on BmNPV replication and proliferation. CRISPR/Cas9-mediated gene knockdown of *BmUba1* resulted in a marked reduction of its protein levels in BmN cells (S3A–S3B Fig), thereby leading to a significant suppression of BmNPV replication and proliferation compared to the control cells (Figs 3A–3C, S3D and S3E). Whereas, overexpression of *BmUba1* had no notable effect on BmNPV proliferation (Figs 3D–3F, S3G and S3H). Subsequently, the impact of *BmUba1* on the antiviral properties of quercetin was investigated. *BmUba1*-knockdown BmN cells infected with BmNPV-EGFP were then treated with 5 μM quercetin-a concentration at which BmNPV replication was not significantly reduced in wild - type BmN cells (Fig 1A). Results showed that treatment with 5 μM quercetin significantly reduced BmNPV replication and proliferation in *BmUba1*-knockdown cells compared to both control groups and the untreated knockdown cells (Figs 3G–3I, S3I and S3J). In contrast, overexpression of *BmUba1* significantly attenuated the anti-BmNPV effect of 10 μM quercetin (Figs 3J–3L, S3K and S3L), further indicating the involvement of BmUba1 in modulating the antiviral activity of quercetin.

Consistently, CRISPR/Cas9-mediated gene knockdown of *BmUba1* in silkworm was conducted, and genomic DNA sequencing analysis revealed a double peak near the sgRNA target site (S3M Fig). Sequencing confirmed that homozygous mutants (*BmUba1*^*-/-*^) were exclusively detected in unhatched eggs and dead larvae (S3N Fig), suggesting that a homozygous mutation in *BmUba1* is lethal. Consequently, we investigated the impact of *BmUba1* knockdown on BmNPV proliferation in the heterozygous mutants (*BmUba1*^*+/-*^). Results demonstrated that viral proliferation and replication were markedly reduced in the fat body of *BmUba1*^+/-^ heterozygous mutants, which harbored a 1-bp insertion (Figs 3M and 3N, S3N and S3O). Moreover, the *BmUba1*^+/-^ mutation significantly extended the survival time of BmNPV-infected larvae, and this protective effect was further enhanced when the larvae were administered with 100 μg quercetin per larva. Log-rank (Mantel-Cox) tests were conducted between WT + ODV+DMSO and *BmUba1*^*+/-*^ + ODV+DMSO groups (*p* = 0.0022), as well as between *BmUba1*^*+/-*^ + ODV+DMSO and *BmUba1*^*+/-*^ + ODV+Quercetin groups (*p* = 0.0001), revealing statistically significant difference (Fig 3O).

The previous study demonstrated that BmNPV actively induces G2/M phase arrest to create a favorable environment for its replication. To further explore the effects of quercetin treatment and *BmUba1* ablation on the cell cycle, we detected the mRNA levels of *B. mori cyclin-dependent kinase 1* (*BmCDK1*) and *B. mori Cyclin B* (*BmCyclin B*), two key regulators of G2/M phase transition that are known to modulate BmNPV replication [33]. The results showed that quercetin treatment had no significant effect on the levels of *BmCDK1* and *BmCyclin B* (S1E Fig). Meanwhile, *BmUba1* knockdown upregulated the mRNA levels of both *BmCDK1* and *BmCyclin B* in BmN cells (S3F Fig).

### Protein structural analysis uncovers an interaction between BmUba1 and quercetin

**Fig 4:**
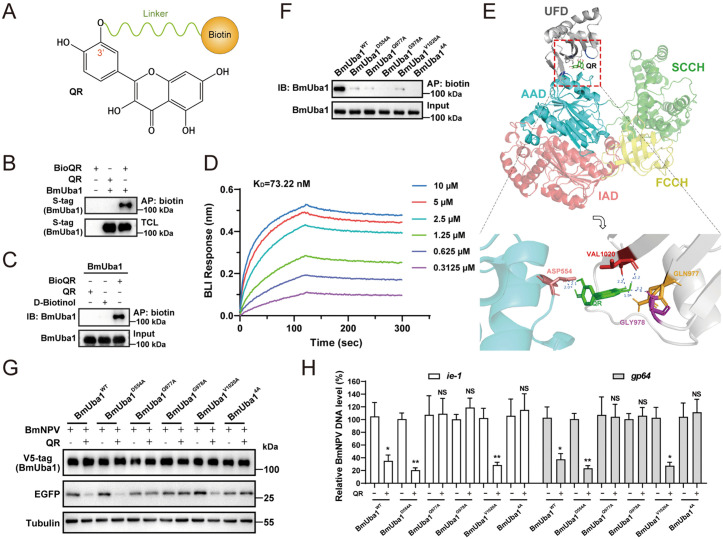
Analysis of the interactions between QR and BmUba1 or its mutant proteins. **(A)** Schematic representation of the biotin-labeled QR probe (BioQR). **(B)** The BmN cells overexpressing *BmUba1-S* were lysed and incubated with the BioQR (14 μM), and then were subjected to affinity pull-down (AP) using Streptavidin Magnetic Beads and immunoblotted with anti-S antibody. **(C)** In direct probe-binding assay, the purified BmUba1 protein (10 μM) was firstly incubated with BioQR (14 μM), and then subjected to AP and immunoblotted with anti-UBA1 antibody. D-biotinol (BIDE, BD00868592) was used as the negative control. **(D)** *In vitro* binding between BioQR and the decreasing concentrations of purified BmUba1 was detected by BLI binding assay. The K_D_ value was the equilibrium dissociation constant. **(E)** Molecular docking simulation with the lowest energy state for the interaction between QR and BmUba1 protein (residues 35-1052). **(F)** Analysis of interaction between BioQR probe and wild-type, single-site mutation of *BmUba1* at D554, Q977, G978, V1020, or its quadruple-site mutation by western blotting. **(G, H)** After a 12-h infection of BmNPV, the *BmUba1*-knockdown cells after overexpressing wild-type and mutant forms of *BmUba1*, were subsequently treated with DMSO or 10 μM QR for 36 h. The cells were collected for measurement of the viral proliferation. Western blot analysis of EGFP protein **(G)**. qPCR analysis of BmNPV *ie-1* and *gp64* **(H)**. Data are presented as mean ± SE (n = 3). Significant differences were evaluated by unpaired Student’s *t*-test **(H)**. *, *p* < 0.05; **, *p* < 0.01; NS, no significance.

To elucidate the precise interaction between BmUba1 and quercetin, we first utilized AlphaFold2 to predict the 3D structure of the BmUba1 protein (S4A Fig). Sequence alignment and structural superposition revealed that BmUba1 consisted of five evolutionarily conserved domains: the inactive and active adenylation domains (IAD and AAD), the first and second catalytic cysteine half-domains (FCCH and SCCH), and the ubiquitin fold domain (UFD). Notably, BmUba1 shared 63.2% sequence identity and exhibited an RMSD (root-mean-square deviation) of 0.954 Å with its mammalian homolog in *Homo sapiens*, as determined by the superposition of 887 out of 984 equivalent Cα atoms (S4B and S4C Fig). Subsequently, the molecular mechanism by which quercetin interacted with BmUba1 to regulate BmNPV proliferation was further investigated. A biotin probe of quercetin was synthesized as previously described (S1 Scheme) [34]. Briefly, biotin was conjugated with quercetin through a tetraethylene glycol chain, and the purity of the final compound was 99.48% as determined by HPLC-MS and NMR (Figs 4A and S5A–S5C). Photo-crosslinking is a biochemical technique that uses UV irradiation (~365 nm) to activate intrinsically photoreactive quercetin, allowing it to form stable in situ covalent bonds with interacting proteins, which we applied to verify the interaction between BmUba1 and biotin-labeled quercetin [35,36]. Notably, total proteins extracted from the lysate of BmN cells overexpressing *BmUba1* bound to the biotin-labeled quercetin, as evidenced by the specific band corresponding to the BmUba1 protein on the blot following photo-crosslinking (Fig 4B). Similarly, the purified BmUba1 protein obtained from a *Escherichia coli* – based prokaryotic expression system was subjected to pull-down assay through incubating with the biotin-labeled quercetin according to the protocol, and the results revealed that BmUba1 bound directly to quercetin *in vitro* (Fig 4C). In addition, the binding affinity between quercetin and BmUba1 protein expressed in *Escherichia coli* was assessed using a BLI (bio-layer interferometry) kinetic binding assay. Results showed that quercetin bound to the BmUba1 protein in a concentration-dependent manner, with fitted K_D_ values calculated to be at 73.22 nM (Fig 4D).

To investigate the interaction mechanism between quercetin and BmUba1, molecular docking of quercetin with BmUba1 was performed using AutoDock Vina software in a semi-flexible docking mode, generating five distinct models (S6A Fig). Based on energy ranking, the model demonstrating the lowest binding energy was chosen for visualization using PyMOL software and further analysis. Notably, quercetin was clearly illustrated in the cartoon depiction, with its binding sites located at the interface between the AAD and UFD within the BmUba1 region. Specifically, the quercetin-binding pocket of BmUba1 is formed by amino acid residues D554 from the α-helices of AAD, Q977 and V1020 from the β-sheets of UFD, and G978 from the loops of UFD, which collectively contributed to the stability of the pocket through the formation of hydrogen bonds (Fig 4E). Accordingly, the four predicted binding sites, D554, Q977, G978, and V1020, were individually mutated, and the corresponding recombinant proteins were expressed and purified from the prokaryotic expression system (S6B Fig). *In vitro* pull-down assay revealed that mutating any one of these four amino acid sites almost completely impaired the binding between BmUba1and quercetin (Fig 4F). Subsequently, to explore the impact of quercetin-binding sites on the antiviral efficacy of quercetin, we performed rescue assays by overexpressing wild-type *Uba1* and various *Uba1* mutants in *BmUba1*-knockdown cells. Results demonstrated that overexpression of wild-type *Uba1*, as well as *Uba1* with mutations at D554 or V1020, allowed quercetin to exert antiviral effects, whereas *Uba1* with mutations at Q977 or G978, or the quadruple mutant, abolished such antiviral activity (Figs 4G–4H and S6C). Furthermore, overexpression of *Uba1* with mutations at Q977 or G978 exerted no obvious effect on the total ubiquitination level in *BmUba1*-knockdown cells (S6D Fig). This indicates that mutation at Q977 or G978 disrupts quercetin binding to the mutated Uba1 instead of impairing its enzymatic activity, thereby abolishing the antiviral effect of quercetin. Thus, Q977 and G978 are critical binding residues for the interaction between quercetin and Uba1 in live cells. Of note, AutoDock docking results indicate that quercetin binds to *Homo sapiens* Uba1 (HsUba1) through specific interactions with residues E557 (AAD domain) and R1032 (UFD domain) (S6E Fig). In summary, BmUba1 physically binds to quercetin through the key sites Q977 and G978, which are crucial for interfering the antiviral effect of quercetin.

### Quercetin inhibits the Ubiquitin-transferring capacity of BmUba1

**Fig 5:**
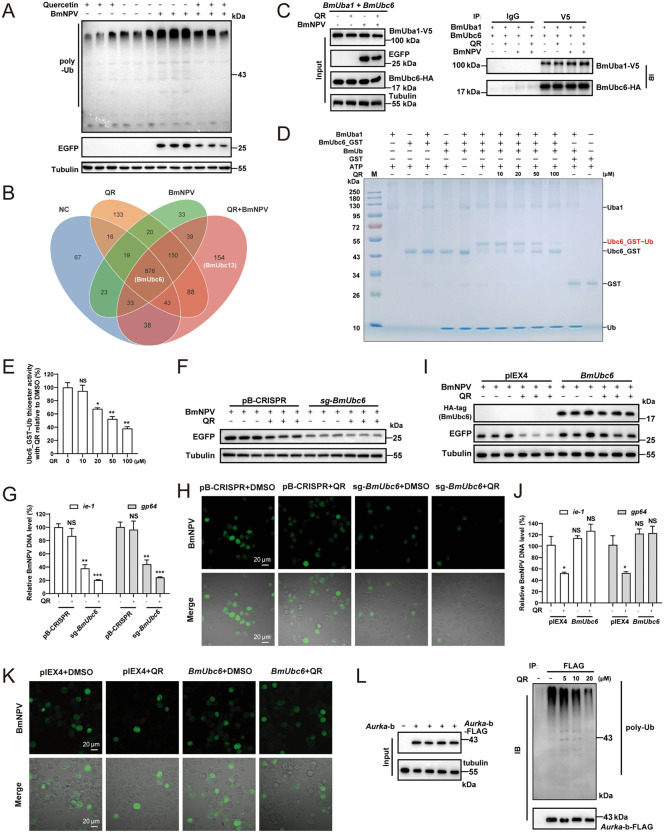
QR inhibits the transfer of BmUb from BmUba1 to BmUbc6. **(A)** After a 12-h infection with BmNPV, BmN cells were treated with DMSO or 10 μM QR for 36 **h.** Western blot analysis of ubiquitination levels of total proteins in BmN cells. **(B)** Venn diagram showing the overlap of immunoprecipitated proteins from the negative control (NC), QR-treated (10 μM), BmNPV (MOI = 5)-infected, and BmNPV + QR groups. **(C)** Co-IP analysis of BmUba1 and BmUbc6 co-overexpressed in BmN cells, which were treated with DMSO, QR, BmNPV, or BmNPV + QR. **(D, E)** An E1-E2 thioester transfer assay was conducted to assess the thioester transfer from BmUba1 to BmUbc6_GST after addition of DMSO or various concentrations of QR (10, 20, 50, and 100 μM) *in vitro*. SDS-PAGE coupled with Coomassie Brilliant Blue staining was performed to determine the level of E2 ~ Ub **(D)**. Quantification of Ubc6_GST ~ Ub bands in D **(E)**. **(F-H)** After a 12-h infection with BmNPV, the *BmUbc6*-knockdown cells were treated with DMSO or 5 μM QR for 36 **h.** Subsequently, the cells were collected for detection of the viral proliferation. Western blot analysis of EGFP protein **(F)**. qPCR analysis of BmNPV *ie-1* and *gp64* **(G)**. Fluorescence observation of GFP protein **(H)**. **(I-K)** After a 12-h infection with BmNPV, the *BmUbc6* overexpressing cells were treated with DMSO or 10 μM QR for 36 **h.** Subsequently, the cells were collected for detection of the viral proliferation. Western blot analysis of EGFP protein **(I)**. qPCR analysis of BmNPV *ie-1* and *gp64* **(J)***.* Fluorescence observation of GFP protein **(K)**. **(L)** After *Aurka-b-FLAG* overexpression for 48 h in BmN cells, then cells were treated with increasing concentrations of QR for 36 **h.** Cell lysates were subjected to immunoprecipitation using the anti-FLAG antibody and immunoblotted with anti-ubiquitin antibody. Data are presented as mean ± SE (n = 3). Significant differences were evaluated by unpaired Student’s *t*-test **(E, G, J)**. *, *p* < 0.05; **, *p* < 0.01; NS, no significance.

Ubiquitination plays a pivotal role in viral replication and proliferation within mammals [37], and we found the overall ubiquitination levels in BmN cells were significantly elevated after BmNPV infection, whereas quercetin effectively counteracted this viral induction, exerting the opposite regulatory effect (Figs 5A and S7A–S7B). The mammalian Uba1, serving as a primary mediator in proteasomal degradation, binds to and activates ubiquitin (Ub), thereby facilitating its transfer to the E2-conjugating Enzyme [38]. Therefore, the E2-conjugating enzyme acting downstream of BmUba1 was initially identified via a screening approach. BmN cells overexpressing *BmUba1* were treated with BmNPV and/or quercetin, and the BmUba1-interacted proteins were immunoprecipitated by the antibody against the BmUba1-fused V5 tag, and then identified by MS. Results revealed that 242 proteins were specifically immunoprecipitated by BmUba1 after BmNPV infection, predominantly annotated in KEGG pathways such as transcription, cancer, and viral infectious diseases (Figs 5B and S7C, S3 and S4 Data). Additionally, 391 proteins were uniquely identified in the quercetin-treated immunoprecipitates of BmUba1, with KEGG annotations indicating their involvement in transcription, neurodegenerative disease, cancer, and environmental adaption (Figs 5B and S7D, S3 and S5 Data). Notably, a total of 154 proteins were specifically immunoprecipitated by BmUba1 in the BmNPV-infected BmN cells treated with quercetin, which were primarily annotated in the pathways including transport and catabolism, folding, sorting and degradation, and neurodegenerative disease (Figs 5B and S7E, S3–S6 Data). Significantly, two distinct E2 proteins were identified in the BmUba1 immunoprecipitates: BmUbc6, which exhibited a consistent binding to BmUba1 across all four groups, and BmUbc13, which only bound to BmUba1 post BmNPV infection followed by quercetin treatment (Fig 5B). Co-IP assay confirmed that BmUba1 interacted with BmUbc6 or BmUbc13 in BmN cells under four different conditions: untreated control, quercetin-treated, BmNPV-infected, and BmNPV-infected followed by quercetin treatment (Figs 5C and S8A).

Molecular docking revealed that the quercetin-binding pocket within BmUba1 was located at the interface of the UFD domain and the AAD domain (Fig 4E). Given that the UFD domain is reported to recruit E2 enzymes and facilitates the E1-E2 thioester transfer, while the AAD domain binds Ub, ATP, and Mg^2+^, exhibiting adenylation activity [39], we further investigated whether the interaction between quercetin and BmUba1 inhibits the thioester transfer of Ub from E1 to E2. First, BmUbc6, BmUbc13, and BmUb proteins were expressed by an *E. coli* expression system and then purified (S8B Fig). Subsequently, the thioester transfer reaction assay was conducted between BmUbc6 or BmUbc13 and BmUb. Results demonstrated that quercetin treatment significantly reduced the conjugation of Ubc6_GST and Ub (Figs 5D and 5E), while the formation of Ubc13_GST ~ Ub remained almost unaffected (S8C and S8D Fig). Furthermore, the thioester formation assay indicated that quercetin treatment did not significantly affect the level of Uba1 ~ Ub (S8E and S8F Fig). Following this, the effects of E2 proteins, BmUbc6 and BmUbc13, on viral proliferation and the antiviral activity of quercetin were investigated. Results showed that *BmUbc6* knockdown significantly suppressed BmNPV proliferation and further enhanced the antiviral activity of quercetin (Figs 5F–5H and S8G–S8I). In comparison, *BmUbc13* knockdown also resulted in a decrease in viral proliferation but did not significantly influence the drug’s efficacy (S9A–S9F Fig). Notably, overexpression of *BmUbc6* did not affect BmNPV proliferation but impaired the antiviral effect of quercetin (Figs 5I-5K, S9G and S9H). In contrast, overexpression of *BmUbc13* had no impact on either viral replication or the antiviral activity of quercetin (S9I–S9M Fig).

Next, we further analyzed the effect of quercetin on the ubiquitination of Ubc6 downstream targets. Based on a previous study [40], we identified and verified *B. mori* Aurora Kinase B (BmAurka-b, BMSK0005274) as a downstream substrate of BmUbc6 via homology analysis using the SilkDB database and immunoprecipitation (S9N Fig). Immunoprecipitation of BmAurka-b-FLAG revealed that BmAurka-b polyubiquitination was obviously reduced following treatment with 10 µM and 20 µM quercetin (Fig 5L). This finding further confirmed that quercetin inhibits ubiquitin transfer from BmUba1 to BmUbc6.

### Quercetin-Uba1 functions conservatively in inhibiting the replication of PRRSV in porcine cells and monkey cells

**Fig 6:**
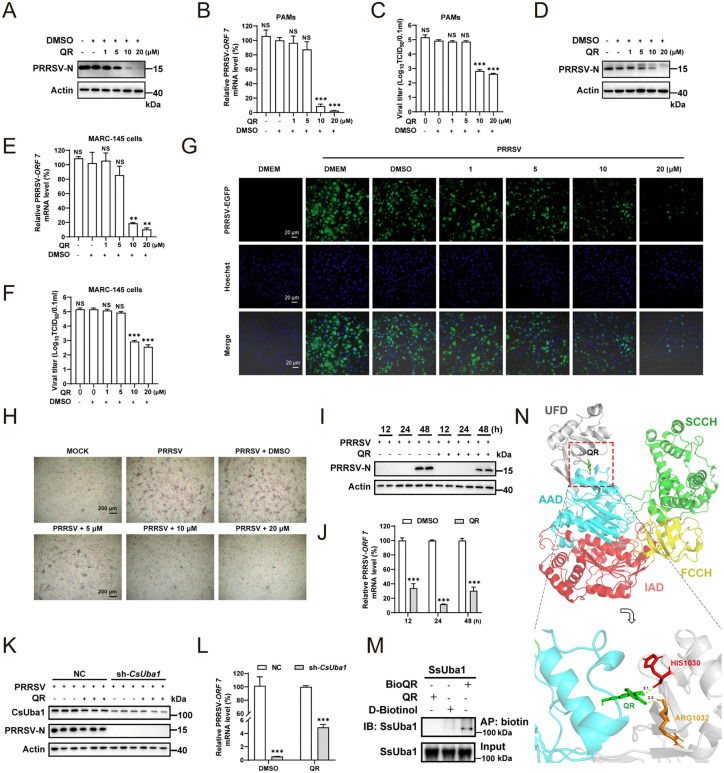
QR inhibits the replication of PRRSV through the interaction with UBA1 homolog in mammals. **(A-C)** After a 12-h infection with PRRSV, PAMs were treated with DMSO or various doses of QR for 36 **h.** Subsequently, PRRSV proliferation was detected in the PAMs. Western blot analysis of PRRSV-N protein **(A)**. mRNA levels of PRRSV *ORF7* detected by qPCR **(B)**. Viral titer of PRRSV detected by TCID_50_ **(C)**. **(D-H)** After a 12-h infection with PRRSV or PRRSV-EGFP, MARC-145 cells were treated with DMSO or various doses of QR for 36 **h.** Subsequently, PRRSV proliferation was detected in the MARC-145 cells. Western blot analysis of PRRSV-N protein **(D)**. qPCR analysis of PRRSV *ORF7* **(E)**. Detection of viral titer of PRRSV by TCID_50_ **(F)**. Fluorescence observation of EGFP protein **(G)**. The cytopathic effect (CPE) in MARC-145 cells after PRRSV infection was observed under an optical microscope **(H)**. **(I, J)** After a 12-h infection with PRRSV, MARC-145 cells were treated with DMSO or 10 μM QR. Subsequently, PRRSV proliferation was detected in the MARC-145 cells at 12 h, 24 h, and 48 h post QR treatment. Western blot analysis of PRRSV-N protein levels **(I)**. qPCR analysis of PRRSV *ORF7* **(J)**. **(K, L)** After a 12-h infection with PRRSV, the *CsUba1*-knockdown cells were treated with DMSO or 5 μM QR for 36 **h.** Subsequently, PRRSV proliferation was detected in the MARC-145 cells. Western blot analysis of PRRSV-N protein **(K)**. qPCR analysis of PRRSV *ORF7* **(L)**. **(M)** In direct probe-binding assay, purified SsUba1 protein (10 μM) incubated with BioQR (14 μM), was subjected to AP and immunoblotted with anti-UBA1 antibody. **(N)** Molecular docking simulation with the lowest energy state for the interaction between QR and SsUba1 protein (residues 49-1058). Data are presented as mean ± SE (n = 3). Significant differences were evaluated by unpaired Student’s *t*-test **(B, C, E, F, J, L)**. *, *p* < 0.05; **, *p* < 0.01; ***, *p* < 0.001; NS, no significance.

Previous studies have demonstrated that quercetin inhibits the replication of RNA virus PRRSV in pigs [4]. Consequently, whether the quercetin-Uba1 axis functioned conservatively in mammals was investigated in the quercetin-PRRSV interaction model. Monitored by PRRSV N protein levels and mRNA levels of PRRSV *ORF7*, it was observed that treatments with gradient doses of quercetin, specifically at 10 μM and 20 μM, markedly suppressed the replication and proliferation of PRRSV in porcine alveolar macrophages (PAMs) (Figs 6A–6C and S10A). Cell survival assay revealed that the CC_50_ of quercetin in PAMs was 202.11 μM. Quercetin exhibited high anti-PRRSV activity across the tested gradient doses without inducing cytotoxicity (S10B Fig). MARC-145 cells (embryonic kidney cells from *Chlorocebus sabaeus*), which are highly susceptible to PRRSV and possess stable subculture ability, are widely used for screening drugs targeting PRRSV; therefore, we further evaluated the antiviral activity of quercetin in this cell line [41]. Similarly, quercetin treatments at concentrations of 10 and 20 μM also exhibited significant inhibitory effects on PRRSV replication (Figs 6D–6G, S10C and S10D), and significantly alleviated the cellular damage of MARC-145 cells caused by PRRSV infection (Fig 6H). Cell survival assay unveiled that the CC_50_ of quercetin was 388.57 μM in MARC-145 cells (S10E Fig). Subsequently, the effect of 10 μM quercetin on PRRSV proliferation was assessed at 12, 24, and 48 h post infection, and results showed that the drug consistently suppressed PRRSV replication in MARC-145 cells (Figs 6I and 6J, S10F). In addition, pretreatment with 10 μM quercetin for 6 h significantly inhibited PRRSV replication in MARC-145 cells (S10G–S10I Fig). SPR-HPLC-MS/MS was employed to screen for proteins that bind to quercetin in *S. scrofa* PAMs, and a total of 170 proteins were identified through MS (S7 Data). KEGG annotation showed that the quercetin-binding proteins in PAM cells were mainly involved in the pathways including signal transduction, cancer: overview, cardiovascular disease, immune system, and endocrine system (S10J Fig), aligning with the pathways identified in silkworms. Heat map analysis of the top 50 proteins captured by quercetin revealed that nearly all of these proteins were the homologs identified in *B. mori*; notably, the six potential target proteins with the highest scores identified in BmN cells, including SF3B3, SERCA, TOP2, xanthine dehydrogenase (XDH), Uba1, and Hsp83, displayed conserved binding properties in PAMs (S10K Fig). To verify the conserved function of Uba1 in the interaction between PRRSV and quercetin, we initially assessed the protein levels of CsUba1 in MARC-145 cells following PRRSV infection for 36 h. Results demonstrated a significant upregulation of CsUba1 protein following the viral infection (S10L and S10M Fig). Of note, the protein levels of CsUba1 were significantly reduced after 12-h PRRSV infection followed by quercetin treatment for 36 h in MARC-145 cells (S10N and S10O Fig). Collectively, these results suggest that, similar to the findings in silkworms, quercetin inhibits viral proliferation in mammalian cells not only by directly interacting with Uba1 but also by downregulating Uba1 expression.

**Fig 7:**
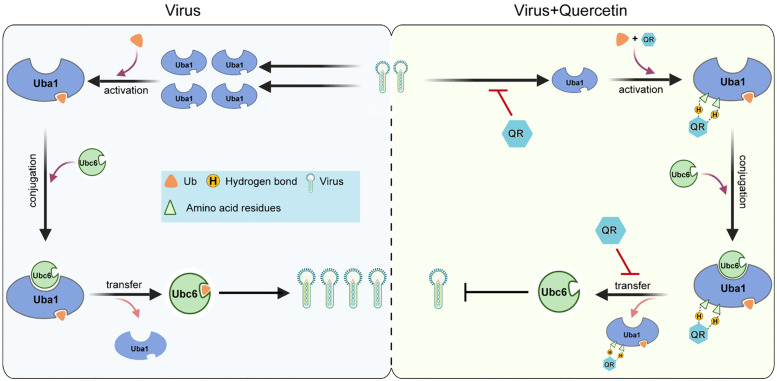
A proposed model illustrating how QR functions the antiviral effects via targeting the ubiquitin-activating system. In virus-infected cells (left), the virus enhances ubiquitination levels by upregulating the protein level of Uba1 (E1, ubiquitin-activating enzyme) and exploits the ubiquitination machinery to facilitate its replication. In QR-treated cells (right), the compound downregulates E1 protein level, while simultaneously binds to E1, and suppresses the transfer of Ub from E1 to Ubc6 (E2, ubiquitin-conjugating enzyme), thereby inhibiting the replication of DNA and RNA viruses. The figure was designed using Biorender. Created in BioRender. Li, H. (2026) https://BioRender.com/9c581sr.

Subsequently, knockdown of *C. sabaeus* Uba1 (*CsUba1*) mediated by shRNA (specific short-hairpin RNA) was performed, and the results showed that *CsUba1* knockdown significantly reduced PRRSV proliferation, whether treated with DMSO or quercetin (Figs 6K and 6L, S10P). To elucidate the interaction between quercetin and *S. scrofa* Uba1 (SsUba1), the purified SsUba1 produced from *E. coli* expression was incubated with the biotin-labeled quercetin, and the pull-down assay indicated that SsUba1 could directly bind to quercetin *in vitro* (Figs 6M and S10Q). Notably, the global ubiquitination levels of proteins in MARC-145 cells were markedly elevated after PRRSV infection, similar to that change observed in BmN cell post BmNPV infection (S10R Fig). Furthermore, molecular docking simulations were conducted using AutoDock Vina software in a semi-flexible docking approach, and the model with the lowest binding energy of -23.01 kJ/mol revealed that SsUba1 interacted with quercetin through residues H1030 and R1032 located at the interface between the UFD and AAD domains (Fig 6N). In summary, quercetin exerts comparable antiviral effects in mammals when responding to mRNA virus PRRSV infection by targeting Uba1, a mechanism that has been evolutionary conserved from insects to mammals (Fig 7).

## Discussion

Quercetin, a flavonoid renowned for its diverse biological activities, has been extensively studied for its multistage antiviral properties in mammals. For instance, it has been reported to block the attachment of severe acute respiratory syndrome coronavirus 2 (SARS-CoV-2) to host cells by interacting with the angiotensin-converting enzyme 2 (ACE2) receptor on the host cell membrane, and reduce viral replication by targeting and inhibiting the activity of 3C-like protease (3CLpro) [42,43]. By binding to the viral glycoprotein G, quercetin also blocks the entry of respiratory syncytial virus (RSV) into host cells [44]. Furthermore, pretreatment with quercetin suppresses the expression of multiple cytokines mediated by NF-κB in human retinal pigment epithelial (ARPE-19) cells, thereby protecting the host from viral infection and inflammatory damage [45]. Furthermore, low-dose quercetin exerts no significant impact on cell cycle progression in normal cells, while strong *Uba1* mutant alleles in *Drosophila* induce cell cycle arrest [46,47]. Our findings demonstrated that pretreatments with quercetin exerted a remarkable inhibitory effect on the replication of both BmNPV and PRRSV, indicating that quercetin not only suppresses viral proliferation after DNA and RNA virus infection, but also exerts substantial protective effects prior to viral infection. In comparison with 5-pyridoxolactone, while quercetin requires a higher working concentration for viral inhibition, 20 μM quercetin showed no significant cytotoxicity, highlighting its favorable safety and promising value for research and development as an anti-BmNPV candidate compound [48]. Of note, we identified Uba1, the ubiquitin-activating enzyme E1, as a key target of quercetin in regulating the replication of BmNPV and PRRSV. Specifically, all four mutations disrupted the in vitro binding between quercetin and BmUba1, only the *BmUba1* with mutations at Q977 or G978 abolished the antiviral effect of quercetin in cells. This discrepancy likely stems from differences in experimental systems: the in vitro binding assay uses purified proteins, whereas the overexpression assay is carried out in living cells, with the two systems varying greatly in molecular environment, protein conformation and cofactor availability. It is particularly noteworthy that quercetin inhibits both DNA (BmNPV) and RNA (PRRSV) viruses by downregulating E1 protein levels, directly binding to E1, and suppressing the transfer of ubiquitin from E1 to Ubc6 (E2, ubiquitin-conjugating enzyme), ultimately inhibiting viral replication (Fig 7). This conserved molecular mechanism highlights quercetin’s broad-spectrum antiviral potential and offers a novel target for developing antiviral drugs against diverse viral infections. Moreover, Treatment with quercetin at working antiviral concentrations exerted no obvious effects on the mRNA levels of *BmCDK1* and *BmCyclin B*, indicating that its antiviral activity is independent of cell cycle regulation. In contrast, *BmUba1* knockdown upregulated the expression of these two factors, implying that *BmUba1* ablation may interfere with virus-induced G2/M arrest and create an unfavorable cellular environment for viral proliferation. Collectively, cell cycle regulation mediated by *Uba1* knockdown may also represent one of the critical pathways for suppressing viral replication, and its underlying mechanism can be explored in greater depth in future investigations.

Ubiquitination is a critical post-translational modification that plays a key role in various cellular processes, including viral infection and replication. Notably, quercetin appears to exert its antiviral activity through multiple mechanisms, particularly by targeting components of ubiquitin-activating system. For example, overexpression of the ubiquitin-activating enzyme (*E1*) downregulates, whereas knockdown of *E1* upregulates, the level of viral capsid protein ICP5, thereby respectively inhibiting or promoting the replication and proliferation of herpes simplex virus type 1 (HSV-1) [49,50]. In this study, BmNPV and PRRSV replication was accompanied by an increased level of Uba1 protein and total protein ubiquitination, while quercetin treatment reversed this effect by reducing Uba1 levels. The modest upregulation of Uba1 observed specifically at 36 hours post-infection likely represents an adaptive host response during the peak of viral replication, a time when enhanced ubiquitination may be required to support viral proliferation. The subsequent reduction in Uba1 levels induced by quercetin at later time points may not stem from its direct binding to Uba1 alone, but rather is an indirect consequence resulting from quercetin’s inhibition of viral replication and associated host pathways. Both the direct binding of quercetin to Uba1 and its indirect downregulation of Uba1 levels collectively contribute to the suppression of BmNPV proliferation. Moreover, knockdown of the *Uba1* homologs enhanced the antiviral effects of quercetin in both insect and mammalian models. As an essential host factor facilitating BmNPV proliferation, knocking down *BmUba1* or *BmUbc6* leads to a reduction in viral levels. Upon subsequent quercetin treatment, the drug can still exert its antiviral effects through the remaining BmUba1, thereby causing a further decrease in viral load.

Currently, there are relatively few reports on the relationship between quercetin and ubiquitination in mammals. We therefore evaluated the conservation of quercetin’s inhibitory effect on Uba1 between *B. mori* and humans. Structural analysis and the docking results of BmUba1–quercetin interaction revealed that the binding sites were located at the interface between the AAD and UFD, spatially distant from the ATP-binding pocket. The ATP-binding pocket of Uba1, which was situated within the AAD domain, was crucial for the adenylation of Ub and the formation of high-energy thioester bonds [39]. The structures of BmUba1 and HsUba1 (PDB: 6DC6) were superimposed with an RMSD value of less than 1 Å, and the interaction sites between HsUba1 and quercetin were similarly situated at the interface of UFD and AAD. The evidence indicates that quercetin is likely to inhibit Ub transfer in humans via an analogous mechanism, providing novel insight into the molecular basis of quercetin’s broad biological activities in humans.

Acting as the initiating enzyme in the ubiquitin conjugation pathway, Uba1 recognizes and activates Ub, then catalyzes its transfer to E2 enzymes [11]. Small molecule compounds usually exert specifically biological activities by influencing the functions of target proteins. Unlike TAK-243, a first-in-class Uba1 inhibitor that interferes with the global ubiquitination process by inhibiting Ub conjugation [51], quercetin selectively inhibited Ub transfer from BmUba1 to BmUbc6, while leaving Ub loading of BmUbc13 was unaffected. This selectivity provides a new direction for developing targeted Uba1 inhibitors.

Notably, Uba1 serves as an essential ubiquitin-activating enzyme that is indispensable for maintaining normal cell viability and host physiological homeostasis. As reported, genetic ablation or complete functional disruption of Uba1 leads to severe developmental defects and even embryonic lethality, which restricts its direct clinical application as an antiviral drug target [52]. Although our study demonstrates that Uba1 mediates the broad-spectrum antiviral activity of quercetin and can be regarded as a valuable mechanistic research target, direct pharmacological blockade or permanent knockout of Uba1 is not clinically feasible. However, the two amino acid sites Q977 and G978, the target of quercetin, do not affect the enzymatic activity of Uba1, making them a potential target for antiviral research.

Beyond its potent antiviral activity, quercetin exhibits other promising bioactivities, including anti-cancer, anti-inflammatory and immune-modulatory effects [53]. These effects are often closely associated with ubiquitination pathways. For instance, dysregulation of ubiquitination and deubiquitination is a hallmark of many cancers, while ubiquitin modifications modulate inflammatory responses by regulating protein stability and complex assembly [54,55]. E3 ligases also help balance immune response and tolerance by degrading key signaling molecules [56]. Although quercetin treatment extended the lifespan of silkworm larvae and alleviated BmNPV-induced midgut damage, its antiviral efficacy was limited and could not prevent larvae death. This may be attributable to the complex physiological environment of the larvae and issues related to quercetin absorption and metabolism [57]; the precise causes warrant further investigation.

## Materials and methods

### Silkworms and cell culture

Silkworms (Nistari and p50 strain) were provided by the Sericultural and Agri-Food Research Institute of the Guangdong Academy of Agricultural Sciences (Guangzhou, China). Larvae were fed with fresh mulberry leaves at 25°C under a 14 h light/10 h dark cycle [58]. BmN cells (ATCC, CRL-8910) were maintained in Grace’s insect medium (Sigma-Aldrich, G9771) supplemented with 10% (v/v) fetal bovine serum (AusGeneX, FBS500-S) at 28°C in an incubator [59]. PAMs and MARC-145 cells were cultured in Dulbecco’s modified Eagle’s medium (Gibco, C11995500BT) supplemented with 10% (v/v) fetal bovine serum (Pricella, 164210) at 37°C in a 5% CO_2_ incubator [60].

### Viral infection and TCID_50_ assay

The recombinant BmNPV-EGFP, the BmNPV budded virion (BV), was generated in our laboratory, and was stored at 4°C in the dark after determination of the 50% tissue culture infectious dose (TCID_50_) [61]. ODV of BmNPV, was provided by the Sericultural and Agri-Food Research Institute of the Guangdong Academy of Agricultural Sciences. For BV infection, BmN cells were incubated with BmNPV-EGFP (MOI = 5) for 1 h, and then replaced with fresh medium; the silkworm larvae were injected with BmNPV-EGFP (5.25 × 10^6^/larva) at day 2 of the fifth instar (5L2D), and the larvae injected with the same volume of virus-free medium were used as controls. Moreover, the silkworm larvae were orally infected with ODV (5.46 × 10^9^/larva) at 5L1D, and the control groups were fed with the equivalent volume of PBS as previously described [62]. PRRSV and recombinant PRRSV-EGFP were kindly provided by Prof. Changxu Song (South China Agricultural University, China). PAMs and MARC-145 cells were incubated with PRRSV or PRRSV-EGFP (MOI = 1) for 1 h, and then replaced with fresh medium. PAMs and MARC-145 cells seeded in 96-well plates were incubated with gradient-diluted PRRSV for 72 h, and the viral titers were subsequently determined by calculating 50% TCID_50_ using the ReedMuench method.

### Quercetin treatment, cytotoxicity and selectivity index detection

Quercetin (aladdin, Q111274) dissolved in DMSO was diluted in Grace’s insect medium before treatment. *In vitro*, following infection with BmNPV-EGFP, PRRSV or PRRSV-EGFP for 1 h, BmN, PAMs and MARC-145 cells were replaced with virus-free medium for 12-h incubation, and then treated with different doses of quercetin (0.5 nM, 1 nM, 500 nM, 1 μM, 2.5 μM, 5 μM, 10 μM or 20 μM) for 36 h. For the drug preincubation experiment, BmN or MARC-145 cells were pre-treated with quercetin (10 μM) for 1 h or 6 h, after which the quercetin-containing medium was completely removed prior to BmNPV-EGFP or PRRSV infection. Subsequent incubation was performed in fresh medium without quercetin, and viral levels were detected at 36 h post-infection. *In vivo*, quercetin stock solutions (4 μg/μL or 20 μg/μL) were diluted 1:1 with insect medium. Afterwards, quercetin (6 μg/larva) was injected into silkworm larvae at the penultimate pair of abdominal legs using a microinjector, at 24 h post-infection with BmNPV-EGFP; larvae were fed with quercetin (100 μg/larva) after ODV oral infection for 24 h; the control groups were treated with an equivalent volume of DMSO using the same administration method.

BmN, PAMs and MARC-145 cells seeded in 96-well plates were incubated with quercetin dissolved in DMSO, at a series of concentrations (2.5 μM, 5 μM, 10 μM, 20 μM, 40 μM, 80 μM, 100 μM, 200 μM and 400 μM) for 48 h, treatment of equivalent volume of DMSO was used as control. Cytotoxicity assessment was conducted using Enhanced Cell Counting Kit-8 (Beyotime, C0042).

BmN cells seeded in 12-well plates were incubated with BmNPV-EGFP for 1 h, after which the virus-containing medium was removed and cells were cultured for another 12 h. Subsequently, the cells were treated with quercetin at concentrations ranging from 2.5 μM to 20 μM for 36 h. The viral fluorescence was observed under a fluorescence microscope, and the 50% inhibitory concentration (IC₅₀) was calculated using the Reed-Muench method [63]. The selectivity index (SI) was defined as the ratio of CC₅₀ to IC₅₀ [64].

### Plasmid construction and transfection

The full-length sequences of *BmUba1*(XM_004923615.4) and its mutants (*BmUba1_D554A, BmUba1_Q977A, BmUba1_G978A*, *BmUba1_V1020A*, and the quadruple-site mutation), along with *BmUbc6* (XM_004931252.5) and *BmUbc13* (NM_001046928.1) were cloned from the total cDNAs of *B. mori* tissue and inserted into the pIEX4 overexpression vector fused with S/V5, HA or FLAG tags, respectively [65]. The primers for gene overexpression were shown in S1 Table. The *B. mori ubiquitin* (*BmUb,* amino acid residues 1–76, AF308163.1), *BmUbc6*, *BmUbc13*, *BmUba1* (amino acid residues 35–1052) and its aforementioned site-specific mutants, and *SsUba1* (amino acid residues 49–1058, XM_013990866.2) were cloned and constructed into the pGEX expression vector, which contained a glutathione S-transferase (GST) tag at the N-terminus, followed by a 3C cleavage site. The primers for recombinant protein expressions were shown in S2 Table. BmN cells were transfected with plasmids using the *Trans*IT-Insect Transfection Reagent (Mirusbio, MIR 6106) according to the manufacturer’s protocol. The cells were replaced with fresh medium at 12 h after transfection, and then treated with BmNPV and/or quercetin for specified time intervals, and finally collected for further analysis.

The shRNA targeting *CsUba1* was synthesized by Tsingke Biotechnology Company (Beijing, China) and inserted into the pLVX-Puro expression vector. The sequence of shRNA was listed in S3 Table. MARC-145 cells were transfected with the plasmids using Lipofectamine 3000 Transfection Reagent (ThermoFisher, L3000008) according to the manufacturer’s protocol.

### Protein expression and purification

The plasmids for recombinant protein expression were transformed into *E. coli* strain BL21 (DE3) through transformation, and used for expressing GST-tagged recombinant proteins as previously described [66]. Briefly, the bacterial cells cultured in LB medium were induced by addition of 0.4 mM Isopropyl β-D-thiogalactoside (IPTG) overnight at 16°C. The expressed proteins were purified using Glutathione Sepharose 4B resin (cytiva life sciences, 17075601), specifically designed for GST-tagged protein purification. Following purification, the GST-tag was removed from the proteins by cleavage with PreScission protease, and the proteins were subsequently separated through gel filtration chromatography, eluting with phosphate-buffered saline (PBS; 140 mM NaCl, 2.7 mM KCl, 10 mM Na_2_HPO_4_, 1.8 mM KH_2_PO_4_, pH 7.4). The protein eluate was centrifuged at 4°C and then resuspended in the buffer composed of 20 mM Tris-Cl pH 8.0, 200 mM NaCl, 300 mM Imidazole, and 5 mM β-ME. The purification process of protein was repeated for 5 times and then concentrated prior to storing at -80 °C.

### Biotin labeling of quercetin and the binding assay

As described in reference [34], a biotin-labeled quercetin probe, specifically the O3’- biotinylated derivative of quercetin (BioQR), was synthesized according to reaction scheme presented in the Supplementary Material, achieving a yield of 50.8%. The final compound underwent comprehensive characterization using high performance liquid chromatography (HPLC), mass spectrometry (MS), and nuclear magnetic resonance (NMR) techniques.

BmN cells overexpressing *BmUba1* for 48 h were collected, and lysed for total proteins using NP-40 buffer (Beyotime, P0013F) supplemented with a protease inhibitor cocktail (Roche, 0469313201). The lysates were centrifuged at 13, 000 × g for 15 min at 4°C, and the supernatant was collected and then incubated with the BioQR on ice, followed by irradiation under a 365 nm UV lamp for 30 min [35]. The prokaryotic-expressed proteins (SsUba1, BmUba1, and its mutants) were directly incubated and cross-linked with BioQR after purification under the UV irradiation. Subseqently, the mixture was incubated with Streptavidin Magnetic Beads (MedChemExpress, HY-K0208) overnight at 4°C, and subjected to immunoprecipitation following the standard procedure. Finally, the reacted beads or cell lysate were mixed with 5 × SDS loading buffer, boiled for 10 minutes, and then submitted to the detection of western blot.

### Co-Immunoprecipitation (Co-IP) and western blot

BmN cells co-overexpressing *BmUba1-V5* and *BmUbc6-HA* or *BmUba1-V5* and *BmUbc13-FLAG* for 48 h were subjected to different treatments: BmNPV infection for 48 h, treatment with quercetin for 36 h following a 12-h BmNPV infection, treatment with quercetin alone for 36 h. The control groups were treated with DMSO and/or Grace’s insect medium. Subsequently, the BmN cells were collected and lysed using NP-40 buffer supplemented with cocktail for 30 min on ice. The supernatant obtained after centrifugation was incubated with an antibody against V5 tag at 4°C for 3 h, and then incubated with Protein A/G Magnetic Beads (MedChemExpress, HY-K0202) at 4°C overnight following the procedure of Co-IP [67,68].

Western blot was performed according to the standard protocol. Briefly, the proteins were separated by SDS-PAGE gel and transferred to PVDF membranes (Millipore, PM-996). Subsequently, the membranes were incubated with the specified primary antibodies, followed by incubation with the corresponding secondary antibodies. The antibodies against Tubulin (1:4000, AT819), SERCA (1:1000, AF6258), Uba1 (1:1000, AF2710), Hsp90 (1:1000, AF7137), Hsp70 (1:1000, AF0189), and Actin (1:3000, AF2811) were purchased from Beyotime Biotechnology; the SF3B3 (1:1000, 14577–1-AP) primary antibody was purchased from Proteintech Group; TOP2 (1:1000, bs-17324R) antibody was purchased from Bioss Biotechnology; S-Tag (1:5000, T0016) antibody was purchased from Affinity Biosciences; GFP (1:4000, HT801) antibody was purchased from TransGen Biotech; PRRSV-N (1:3000, GTX129270) antibody was purchased from GeneTex Biotech; V5-Tag (1:200, 13202S), HA-Tag (1:200, 3724S), and FLAG-Tag (1:200, 14793S) antibodies were purchased from Cell Signaling Technology. Finally, protein bands were observed under Chemiluminescent Images System (Tanon-5200) as previously described [67]. The Tubulin and Actin were used as the reference proteins. Quantification of western blots was analyzed using ImageJ software.

### Quantitative PCR (qPCR)

Total RNA was extracted from fat body, BmN, PAMs, and MARC-145 cells by TRIzol reagent (Vazyme, R411), and cDNA was synthesized using the reverse transcription kit (Takara, RR037A). Total DNA was isolated from fat body and BmN cells by the insect genome extraction kit (Magen, D3129). qPCR was performed using the Universal SYBR qPCR Master Mix (Vazyme, Q711) according to the manufacturer’s instructions. The data were collected by Bio-Rad CFX Manager software (version 3.0), and analyzed by the 2^−∆∆Ct^ method as previously described [69]. The *ie-1* and *gp64* genes of BmNPV and *ORF7* (nucleocapsid) of PRRSV were used for indicating the level of virus replication, and *BmGAPDH, Bmrp49* and *mammalian actin* were used as the reference genes. The primers for qPCR were listed in S4 Table.

### Bio-layer interferometry (BLI) analysis

BLI analysis was performed to assess the binding kinetics between protein and small molecules using the Octet RED96e System (ForteBio) [70]. Initially, BioQR was immobilised onto the Streptavidin biosensors (ForteBio, 18–5019) pre-wetted with PBS for 5 min. Subsequently, the biosensors were equilibrated with the Kbuffer (10 mM PBS, 1% DMSO, pH 7.4) to remove any unbound BioQR. Next, the biosensor tips were inserted into the sample plate containing protein solution of various concentrations, which had been diluted with the Kbuffer. The tips were then incubated in these protein solutions for 5 min. Finally, protein dissociation from BioQR was achieved by washing the biosensor with Kbuffer for 5 min. The entire experimental procedure was carried out at a temperature maintained below 25°C. The signal data were processed and analyzed using Octet software (version 11.0, ForteBio), applying the 1:1 binding model with a global fitting approach.

### CRISPR/Cas9-mediated gene knockdown

CRISPR/Cas9 technology was employed for the gene knockdown in BmN cells [23]. sgRNA of target genes were designed online (http://crispor.tefor.net/) and inserted into the pB-CRISPR vector, which was subsequently co-transferred with A3-Helper plasmid in BmN cells using the TransIT-Insect Transfection Reagent (Mirus Biotech, WI, USA, MIR6100). After transfection for 48 h, the BmN cells were subjected to a selective culture with the addition of Zeocin Selection Reagent (200 μg/mL, Invivogen, ant-zn-1p) for about one month to eliminate those cells that failed to integrate the CRISPR/Cas9 system into the genomes [30]. Subsequently, the whole genome of the knockdown cells was extracted using Genomic DNA Kit (Tiangen, DP316), and DNA fragment containing sgRNA target site was amplified to evaluate the knockdown efficiency by sequencing. The primers for sgRNA were shown in S5 Table.

For *BmUba1* knockdown in *B. mori* larvae *in vivo*, a double-stranded DNA fragment containing the sgRNA of *BmUba1* and T7 promoter was amplified using the primers (*BmUba1*-T7-F and sgRNA-R), and inserted into the 19T vector (TaKaRa, 6013). Subsequently, the DNA fragment of *BmUba1* SgRNA was amplified from the 19T vector using the primers (19 T-F and sgRNA-R). Generation of sgRNA was conducted using Transcript Aid T7 High Yield Transcription Kit (Thermo Scientific, K0441) according to the manufacturer’s instruction. The mixture of sgRNA (at a concentration of 600 ng/μL) and Cas9 protein (400 ng/μL; PNA bio, CP01) were injected into freshly laid silkworm eggs within 4 h by a micro-injector (FemtoJet, Germany). Subsequently, the eggs were incubated at 26°C until the larvae hatched, resulting in the G0 generation [71]. Genotype sequencing of the G0 generation was conducted to identify mutant individuals at adult stage of silkworm. These mutants were then crossed with wild-type silkworms to produce the G1 generation. Likewise, homozygous and heterozygous individuals among the G1 mutants were identified through genotype sequencing, and the heterozygous G1 line was self-crossed [23]. The primers for *BmUba1* knockdown *in vivo* were shown in S6 Table.

### Analysis of quercetin-binding proteins using surface plasmon resonance (SPR)

SPR combined with high-performance liquid chromatography mass spectrometry (HPLC-MS) was employed to identify the quercetin-interacting proteins in both silkworms and pigs. A 10 mM quercetin solution was printed on a 3D photocross-linking sensor chip to serve as a stationary phase using a BioDot-1520 array printer. BmN cells infected by BmNPV for 48 h or PAMs infected by PRRSV for 48 h were harvested and centrifuged at 2,500 × g for 4 min. The cell pellets were then lysed to obtain total proteins, which were subsequently diluted to a final concentration of 200 μg/mL. The protein lysate, acting as the mobile phase, was allowed to bind to the surface of the sensor chip. After that, the chip was washed to remove non-specifically interacted proteins. Finally, the proteins bound to the chip’s surface were digested *in situ* and subjected to protein identification via HPLC-MS [15]. The proteins captured through SPR underwent additional analysis including KEGG annotation and heatmap visualization.

### Immunoprecipitation and mass spectrum analysis

Three groups of *BmUba1* overexpressing BmN cells, infected with BmNPV for 48 h, quercetin treatment for 36 h following 12-h BmNPV infection, quercetin treatment alone for 36 h, were collected and subjected to immunoprecipitation using an antibody against V5 tag and Protein A/G Magnetic Beads, following the standard protocol [61]. Additionally, BmN cells overexpressing *BmUba1* without any treatment served as the control group. The immunoprecipitated proteins underwent digestion with a trypsin solution and were subsequently loaded onto a nanoViper C18 (Acclaim PepMap 100, 75 μm × 2 cm) trap column. Online chromatography separation was conducted using the Easy nLC 1200 system (ThermoFisher). Following the trapping and desalting steps of the peptide fractions, an elution gradient was applied on an analytical column (PepMap RSLC, 75μm × 25 cm C18-2 μm 100 Å). Tandem mass spectrometry (MS/MS) data were acquired using data-dependent acquisition (DDA) mode on a Thermo Fisher Scientific Fusion mass spectrometer (Thermo Fisher Scientific, USA), which was fitted with a Nano Flex ion source. Protein identification and quantification from the MS/MS data were performed using PEAKS Online software through searching against the UniProt (*B. mori*) database.

### Structure prediction and molecular docking

To analyze the interaction between quercetin and the target proteins, the structure of BmUba1 was predicted using the AlphaFold2 framework within the ColabFold [72], and the model with the highest pLDDT score was selected for the subsequent molecular docking studies. The structure of SsUba1 proteins was predicted using homology modeling via the SWISS-MODEL server. Molecular dockings simulations were performed to explore the binding between quercetin and BmUba1, SsUba1, or HsUba1. These simulations were carried out using AutoDock vina molecular docking software (version 3.0.5), which was designed to simulate the binding of proteins to small molecules [73]. The resulting docking models were ranked based on their binding energy values. The domains of BmUba1, SsUba1, and CsUba1 annotated through protein sequence alignments, as well as relevant protein structures, were achieved using PyMOL software (version 2.4).

### Thioester formation and transfer assays

The thioester formation assay was initiated by incorporating a reaction mixture containing 0.5 μM BmUba1, 5 μM BmUb, 10 mM MgCl_2_, 10 mM ATP, 50 mM Tris pH7.5, 25 mM NaCl, and 0.6 mM DTT. Subsequently, the assay was advanced by adding 1 μM of either BmUbc6 or BmUbc13 into the mixture [74]. All assays were conducted at 37°C for a duration of 5 min. Reactions were initiated through the addition of ATP and terminated by the addition of non-reducing sample loading buffer of SDS-PAGE. The samples were then subjected to SDS-PAGE separation at a constant voltage of 120 V for 60 min at room temperature. After separation, the gels were stained with Coomassie Brilliant Blue and observed.

### Bioinformatics analysis

Omicshare online tools were employed for the KEGG pathway annotation and intersection analysis of datasets. Heatmap analysis of mass spectrometry data was performed using the Heml software (Heat map Illustrator, version 1.0). Amino acid sequence alignment was conducted by Clustalx software (version 2.1), and the result was visualized using the online ESPript software. UCSF Chimera software was utilized to perform structural superposition of proteins, enabling comparative analysis.

### Statistical analysis

The experimental data, derived from at least three independent replicates, were presented as the mean ± SE. Before performing Student’s *t*-test and analysis of variance (ANOVA), normality and homogeneity of variance tests were conducted on all datasets to guarantee the validity of the statistical analyses. For the quantitative analysis of viral EGFP fluorescence, measurements were taken on approximately 200 BmN or MARC-145 cells across three biological replicates. Statistical significance was defined as *p* < 0.05 (*), *p* < 0.01 (**), and *p* < 0.001(***), while NS means no significant difference. The two-tailed, unpaired Student’s *t*-test, and one-way ANOVA were carried ou*t* using IBM SPSS software (version 27.0.1.0). The log-rank (Mantel-Cox) test was employed for the statistical analysis of survival curves. The results of these analyses were presented using GraphPad Prism software (version 8.4.2). Each treated group for the survival curves comprised three independent subgroups, with each subgroup containing 15 larvae.

## Supporting information

S1 Fig Statistical analysis of QR’s inhibitory effect on BmNPV proliferation. (A) Quantification of EGFP protein in (Fig 1A). (B) Quantification of EGFP-positive cells in (Fig 1C). (C) Cytotoxicity of QR measured by CCK-8 assay in BmN cells. (D) The effect of gradient doses of QR on BmNPV-EGFP proliferation. (E) Detection of BmCDK1 and BmCyclin B mRNA levels by qPCR. BmN cells were treated with DMSO or gradient doses of QR for 36 h. (F) Quantification of EGFP protein in (Fig 1D). (G) Quantification of EGFP-positive cells in (Fig 1F). (H) Quantification of EGFP protein in (Fig 1G). (I) Quantification of EGFP-positive cells in (Fig 1I). (J) Quantification of EGFP protein in (Fig 1J). (K) Quantification of EGFP protein in (Fig 1M). Data are presented as mean ± SE (n = 3). Statistical significance of differences was assessed using an unpaired Student’s t-test (A-C, E-K). *, p < 0.05; **, p < 0.01; ***, p < 0.001; NS, no significance.
(TIF)

S2 Fig Screening the QR binding targets by SPR in BmN cells. (A) Top 20 enriched pathways of proteins captured by QR molecule. (B) Heatmap of the QR-captured proteins following normalization by their scores and relative quantities. (C and D) Quantification of the six protein levels in (Fig 2C) and (Fig 2D). (E) Western blot analysis of BmUba1 after overexpression in BmN cells. Data are presented as mean ± SE (n = 3). Significant differences were evaluated by unpaired Student’s *t*-test (C, D). *, *p* < 0.05; ***, *p* < 0.001; ns, no significance.
(TIF)

S3 Fig Statistical analysis of the effect of BmUba1 on BmNPV replication and QR’s antiviral activity. (A) Partial genome sequence of *BmUba1* containing the knockdown site and the knockdown efficiency. (B and C) Western blotting of BmUba1 in the *BmUba1*-knockdown cells (B). Quantification of BmUba1 proteins in B (C). (D) Quantification of EGFP proteins in (Fig 3A). (E) Quantification of EGFP-positive cells in (Fig 3C). (F) Detection of mRNA levels of *BmCDK1* and *BmCyclin B* by qPCR in *BmUba1*-knockdown BmN cells. (G) Quantification of EGFP proteins in (Fig 3D). (H) Quantification of EGFP-positive cells in (Fig 3F). (I) Quantification of EGFP proteins in (Fig 3G). (J) Quantification of EGFP-positive cells in (Fig 3I). (K) Quantification of EGFP proteins in (Fig 3J). (L) Quantification of EGFP-positive cells in (Fig 3L). (M) Sequence alignment of mutated *BmUba1* with the WT genomic sequence. (N) Sequencing results of *BmUba1* in the *BmUba1* homozygous mutant. (O) Quantification of EGFP proteins in (Fig 3M). Data are presented as mean ± SE (n = 3). WT means wild type. Significant differences were evaluated by unpaired Student’s *t*-test (C-H, O). *, *p* < 0.05; **, *p* < 0.01; ***, *p* < 0.001; NS, no significance. Statistical differences were evaluated by one-way ANOVA (I-L).
(TIF)

S4 Fig Predicated protein structures of BmUba1 and its comparison with HsUba1. (A) BmUba1 was visualized as a surface-rendered model, with distinct structural domains color-coded for clarity. The binding pockets for QR and ATP were specifically highlighted in blue and magenta, respectively, within a delineated red box. (B) The predicted BmUba1 structure was globally superimposed onto the human Uba1 (HsUba1), with the RMSD value indicated below the structures. (C) Alignment of BmUba1 and HsUba1, with amino acid residues involved in QR interactions marked by red asterisks (BmUba1) and blue asterisks (HsUba1), respectively. The ATP-binding pocket of BmUba1 is indicated in green.
(TIF)

S5 Fig Identification of the biotin-labeled QR. (A) HPLC analysis of biotin-labeled QR (BioQR) purity. (B) MS analysis of molecular weight for BioQR. LC-MS (MS, ESI): m/z 704.20 (M + H). (C) NMR identification of BioQR. ^1^H NMR (400 MHz, DMSO-*d*_6_) δ 12.46 (s, 1H), 10.76 (s, 1H), 9.74 (s, 1H), 9.42 (s, 1H), 7.81 (t, *J* = 5.6 Hz, 1H), 7.77 (d, *J* = 2.0 Hz, 1H), 7.70 (dd, *J* = 8.4, 2.0 Hz, 1H), 6.96 (d, *J* = 8.4 Hz, 1H), 6.48 (d, *J* = 2.0 Hz, 1H), 6.40 (s, 1H), 6.35 (s, 1H), 6.19 (d, *J* = 2.0 Hz, 1H), 4.33-4.25 (m, 1H), 4.18-4.07 (m, 3H), 3.82-3.75 (m, 2H), 3.66-3.59 (m, 2H), 3.58-3.47 (m, 6H), 3.38 (t, *J* = 6.0 Hz, 2H), 3.22-3.13 (m, 2H), 3.12-3.03 (m, 1H), 2.85-2.76 (m, 1H), 2.57-2.50 (m, 1H), 2.05 (t, *J* = 7.2 Hz, 2H), 1.62-1.58 (m, 1H), 1.53-1.37 (m, 3H), 1.35-1.23 (m, 2H).
(TIF)

S6 Fig Analysis of the interactions between QR and BmUba1 or HsUba1. (A) Top 5 models of Molecular Docking between BmUba1 and QR. (B) SDS-PAGE analysis of the wild-type and mutant BmUba1 proteins purified by gel filtration chromatography. (C) Quantification of EGFP proteins in (Fig 4G). (D) *BmUba1*-knockdown cells were transfected with plasmids encoding wild-type *BmUba1* or its Q977 and G978 mutants. The total ubiquitination levels were detected by western blot. (E) The optimal HsUba1-QR docking conformation, characterized by a binding energy of -26.02 kJ/mol, was selected for presentation. Molecular interaction analysis highlighted E557 and R1032 as critical interaction sites. Data are presented as mean ± SE (n = 3). Statistical differences were evaluated by one-way ANOVA (C).
(TIF)

S7 Fig Ubiquitination and KEGG pathway analysis of BmUba1-interacting proteins. (A and B) Western blot analysis of ubiquitination levels of total proteins in BmN cells at 12, 24, 36, and 48 h post BmNPV infection (A) or QR treatment (B). (C-E) KEGG annotation of proteins specifically immunoprecipitated by BmUba1-V5 following BmNPV (C) or 10 μM QR (D) treatment, or BmNPV infection followed by QR treatment (E).
(TIF)

S8 Fig Effects of QR on BmUba1-mediated ubiquitin activation and transfer efficiency. (A) Co-IP assay of *BmUba1* and *BmUbc13* in BmN cells after DMSO, QR (10 μM), BmNPV (MOI = 5), or QR (10 μM) + BmNPV (MOI = 5) treatment. (B) SDS-PAGE detection of BmUb, GST, BmUbc6_GST, and BmUbc13_GST proteins purified by gel filtration chromatography. (C and D) An E1-E2 thioester transfer assay was conducted to evaluate the effect of QR on ubiquitin transfer from BmUba1 (E1) to BmUbc13_GST (E2). The assay was performed in the presence of DMSO control or varying QR concentrations (10, 20, 50, and 100 μM). Ubc13_GST ~ Ub conjugate was detected by SDS-PAGE (C). Quantification of the E2 ~ Ub bands in (C) was shown in (D). (E and F) A thioester formation assay was performed between BmUba1 and Ub after treatments with DMSO or varying concentrations of QR (10, 20, 50, and 100 μM). The level of Uba1 ~ Ub was detected by SDS-PAGE (E). Quantification of the Uba1 ~ Ub bands in (E) was shown in (F). (G) Partial genome sequence of *BmUbc6* containing the knockdown site and the knockdown efficiency. (H) Quantification of EGFP proteins in (Fig 5F). (I) Quantification of EGFP-positive cells in (Fig 5H). Data are presented as mean ± SE (n = 3). Significant differences were evaluated by unpaired Student’s *t*-test (D, F). *, *p* < 0.05; **, *p* < 0.01; ***, *p* < 0.001; NS, no significance. Statistical differences were evaluated by one-way ANOVA (H, I).
(TIF)

S9 Fig Effects of BmUbc6 and BmUbc13 on BmNPV proliferation and QR’s antiviral activity. (A) Partial genome sequence of *BmUbc13* containing the knockdown site and the knockdown efficiency. (B-F) At 12 h post BmNPV infection, *BmUbc13*-knockdown cells were exposed to 5 μM QR or DMSO for 36 h. Viral replication levels were subsequently detected. Western blotting of EGFP protein (B) and its quantification (C). qPCR analysis of viral *ie-1* and *gp64* genes (D). Fluorescence observation of GFP proteins (E) and the quantification of EGFP-positive cells (F). (G) Quantification of EGFP proteins in (Fig 5I). (H) Quantification of EGFP-positive cells in (Fig 5K). (I-M) After a 12-h infection with BmNPV, the *BmUbc13* overexpressing BmN cells were treated with 5 μM QR or DMSO for 36 h. The viral proliferation in BmN cells was subsequently detected. Western blotting of EGFP protein (I) and its quantification (J). qPCR analysis of BmNPV *ie-1* and *gp64* (K). Fluorescence observation of GFP proteins (L) and the quantification of EGFP-positive cells (M). (N) IP assay of *BmUbc6* and *BmAurka-b* in BmN cells after co-transfection for 48 h. Data are presented as mean ± SE (n = 3). Significant differences were evaluated by unpaired Student’s *t*-test (D, K). *, *p* < 0.05; **, *p* < 0.01; ***, *p* < 0.001; NS, no significance. Statistical differences were evaluated by one-way ANOVA (C, F, G, H, J, M).
(TIF)

S10 Fig Effects of QR on PRRSV proliferation. (A) Quantification of PRRSV-N protein in (Fig 6A). (B) Cytotoxicity of QR was measured by CCK-8 assay in PAMs. (C) Quantification of PRRSV-N protein in (Fig 6D). (D) Quantification of EGFP-positive cells in (Fig 6G). (E) Cytotoxicity of QR was measured by CCK-8 assay in MARC-145 cells. (F) Quantification of PRRSV-N protein in (Fig 6I). (G-I) MARC-145 cells were pre-treated with 10 μM QR for 6 h before PRRSV infection (-6 h). The viral proliferation was further detected. Western blotting of PRRSV-N protein (G), and its quantification (H). qPCR analysis of PRRSV *ORF7* mRNA (I). (J) KEGG pathway annotation of proteins captured by QR molecule in PAMs. (K) Heatmap of the captured proteins after normalization by their scores and relative quantities. (L and M) Western blotting of CsUba1 in MARC-145 cells at 36 h post PRRSV infection (L), and its quantification (M). (N and O) Western blotting of CsUba1 (N) in MARC-145 cells after 12 h PRRSV infection followed by 10 μM QR treatment for 36 h, and its quantification (O). (P) Quantification of PRRSV-N protein in (Fig 6K). (Q) SDS-PAGE coupled with Coomassie Brilliant Blue staining of SsUba1 protein purified by gel filtration chromatography. (R) Western blotting of ubiquitination levels of total proteins in MARC-145 cells at 12, 24, 36, and 48 h post PRRSV infection. Data are presented as mean ± SE (n = 3). Significant differences were evaluated by unpaired Student’s *t*-test (A-F, H, I, M, O). *, *p* < 0.05; **, *p* < 0.01; ***, *p* < 0.001; NS, no significance. Statistical differences were evaluated by one-way ANOVA (P).
(TIF)

S1 Table Primers for gene overexpression. (PDF)

S2 Table Primers for recombinant protein expressions. (PDF)

S3 Table Sequences of shRNA. (PDF)

S4 Table Primers for qPCR. (PDF)

S5 Table Primers for sgRNA. (PDF)

S6 Table Primers for *BmUba1* knockdown *in vivo.* (PDF)

S1 Scheme Synthesis route of QR probe. (PDF)

S1 Data List of drugs tested for anti-BmNPV activity. (XLS)

S2 Data List of QR-captured proteins in BmN cells following BmNPV treatment. (XLS)

S3 Data BmUba1-V5-immunoprecipitated proteins (V-Q-). (XLS)

S4 Data BmUba1-V5-immunoprecipitated proteins (V+Q-). (XLS)

S5 Data BmUba1-V5-immunoprecipitated proteins (V-Q+). (XLS)

S6 Data BmUba1-V5-immunoprecipitated proteins (V+Q+). (XLS)

S7 Data List of QR-captured proteins in PAMs following PRRSV treatment. (XLS)

S1 Raw Images Source data file including original uncropped and unadjusted images underlying all blot and gel results. (PDF)
