# Supplementary material for: Quercetin, a flavonoid, suppresses viral proliferation by interfering with the ubiquitin transfer from E1 to E2 enzymes
Source: PLoS Pathog. 2026 Jul 20;22(7):e1014425. doi: 10.1371/journal.ppat.1014425 (PMC13399506; doi:10.1371/journal.ppat.1014425)
Supplement: S1 Table — (PDF) [file ppat.1014425.s011.pdf]

Genes	Forward 5'-3'	Reverse 5'-3'
BmUba1-	ATGTCTAGTGCTG	GCTGTCCATGTGCTGGCGTTCGAATTTAGCAGCAG
S tag	AAGTCGCCG	CGGTTTCTTTAGGCAGAGTGTATTTTACATAC
BmUba1-V	ATGTCTAGTGCTG	CGTAGAATCGAGACCGAGGAGAGGGTTAGGGATA
5 tag	AAGTCGCCG	GGCTTACCAGGCAGAGTGTATTTTACATAC
BmUbc6-H	ATGTCAACTCCAG	AGCGTAATCTGGAACATCGTATGGGTAATCAATAA
A tag	CAAGAAGAC	ATGATTGTTC
BmUbc13-F	ATGGCAGCCCTAC	CTTGTCATCGTCGTCCTTGTAGTCGTTGTCCATGG
LAG tag	CACGTAGAA	CGTATCTCCGC
BmAurka-b	ATGCCTACGGACA	CTTGTCATCGTCGTCCTTGTAGTCTTTTTGCTCAGC
-FLAG tag	GTTTCAAAATCC	ATTCATTAATG

Note: the sequences of multiple tags were marked in red
