# Supplementary material for: Quercetin, a flavonoid, suppresses viral proliferation by interfering with the ubiquitin transfer from E1 to E2 enzymes
Source: PLoS Pathog. 2026 Jul 20;22(7):e1014425. doi: 10.1371/journal.ppat.1014425 (PMC13399506; doi:10.1371/journal.ppat.1014425)
Supplement: S2 Table — (PDF) [file ppat.1014425.s012.pdf]

Genes	Forward 5'-3'	Reverse 5'-3'
BmUba1	GATGAAATCGACGAGAGTCTGT	CTAAGGCAGAGTGTATTTTACATACG
BmUbc6	ATGTCAACTCCAGCAAGA	CTAATCAATAAATGATTGTTCTACAC
BmUbc13	ATGGCAGCCCTACCAC	TCAGTTGTCCATGGCGTATCTCC
BmUb	ATGCAAATTTTCGTAAAGAC	TTAGCCACCACGAAGCCT
SsUba1	GACATAGATGAGGGCCTTTA	TCAGCGGATGGTGTATCGTA
