# Supplementary material for: Quercetin, a flavonoid, suppresses viral proliferation by interfering with the ubiquitin transfer from E1 to E2 enzymes
Source: PLoS Pathog. 2026 Jul 20;22(7):e1014425. doi: 10.1371/journal.ppat.1014425 (PMC13399506; doi:10.1371/journal.ppat.1014425)
Supplement: S3 Table — (PDF) [file ppat.1014425.s013.pdf]

Gene	5'-3'
CsUba1	GATCGCCTCCAACTTCTCCGACTATACTCGAGTATAGTCGGAGAAGTTGGAGGTTTTTT
Negative control (NC)	GATCGACTACCGTTGTTATAGGTGTCTCGAGACACCTATAACAACGGTAGTTTTTTT
