# Supplementary material for: Quercetin, a flavonoid, suppresses viral proliferation by interfering with the ubiquitin transfer from E1 to E2 enzymes
Source: PLoS Pathog. 2026 Jul 20;22(7):e1014425. doi: 10.1371/journal.ppat.1014425 (PMC13399506; doi:10.1371/journal.ppat.1014425)
Supplement: S4 Table — (PDF) [file ppat.1014425.s014.pdf]

Genes	Forward 5'-3'	Reverse 5'-3'
ie1	CGTGAGCAATGTGGTGTACG	AATTTTGTGCAGCCGTCTCG
gp64	ACACGTGCAACAAATCGTGG	ATACTCACGCCGTCTCGATG
BmGAPDH	CATTCCGCGTCCCTGTTGCTAAT	GCTGCCTCCTTGACCTTTTGC
actin	TCTTCCAGCCCTCCTTCTTG	GAGGCGCGATGATCTTGATC
ORF7	GAGAAGCCCCATTTCCCTCT	TGCTGAGGGTGATGCTGTAA
BmCyclin B	GGGAAAGGTAATGGAGCCGT	TACAGCTCTGGCGGTCAATC
BmCDK1	AGGGCTCCTGAGGTCTTACT	TGTTGGCGTTCTTAGCATTCT
Bmrp49	CAGGCGGTTCAAGGGTCAATAC	TGCTGGGCTCTTTCCACGA
