# Supplementary material for: Quercetin, a flavonoid, suppresses viral proliferation by interfering with the ubiquitin transfer from E1 to E2 enzymes
Source: PLoS Pathog. 2026 Jul 20;22(7):e1014425. doi: 10.1371/journal.ppat.1014425 (PMC13399506; doi:10.1371/journal.ppat.1014425)
Supplement: S5 Table — (PDF) [file ppat.1014425.s015.pdf]

Gene	Forward 5'-3'	Reverse 5'-3'
BmUba1	aagtCGATAATTCCGTTGACCCCC	aaacGGGGGTCAACGGAATTATCG
BmUbc6	aagtCGTACTGTTGGGGGTTTATT	aaacAATAAACCCCCAACAGTACG
BmUbc13	aagtCTAGGTACTGCGCTGATCCC	aaacGGGATCAGCGCAGTACCTAG

Note: aagt and aaac are the endonuclease sites of the pB-CRISPR vector
