# Supplementary material for: Quercetin, a flavonoid, suppresses viral proliferation by interfering with the ubiquitin transfer from E1 to E2 enzymes
Source: PLoS Pathog. 2026 Jul 20;22(7):e1014425. doi: 10.1371/journal.ppat.1014425 (PMC13399506; doi:10.1371/journal.ppat.1014425)
Supplement: S6 Table — (PDF) [file ppat.1014425.s016.pdf]

Primers	Sequences 5'-3'
BmUba1-T7-F (Forward)	TAATACGACTCACTATAGGCGATAATTCCGTTGACCCCC
sgRNA-R (Reverse)	AAGCACCGACTCGGTGCC
19T-F (Forward)	CGGTGATGACGGTGAAAACCTC
