# Supplementary material for: Quercetin, a flavonoid, suppresses viral proliferation by interfering with the ubiquitin transfer from E1 to E2 enzymes
Source: PLoS Pathog. 2026 Jul 20;22(7):e1014425. doi: 10.1371/journal.ppat.1014425 (PMC13399506; doi:10.1371/journal.ppat.1014425)
Supplement: S1 Scheme — (PDF) [file ppat.1014425.s017.pdf]

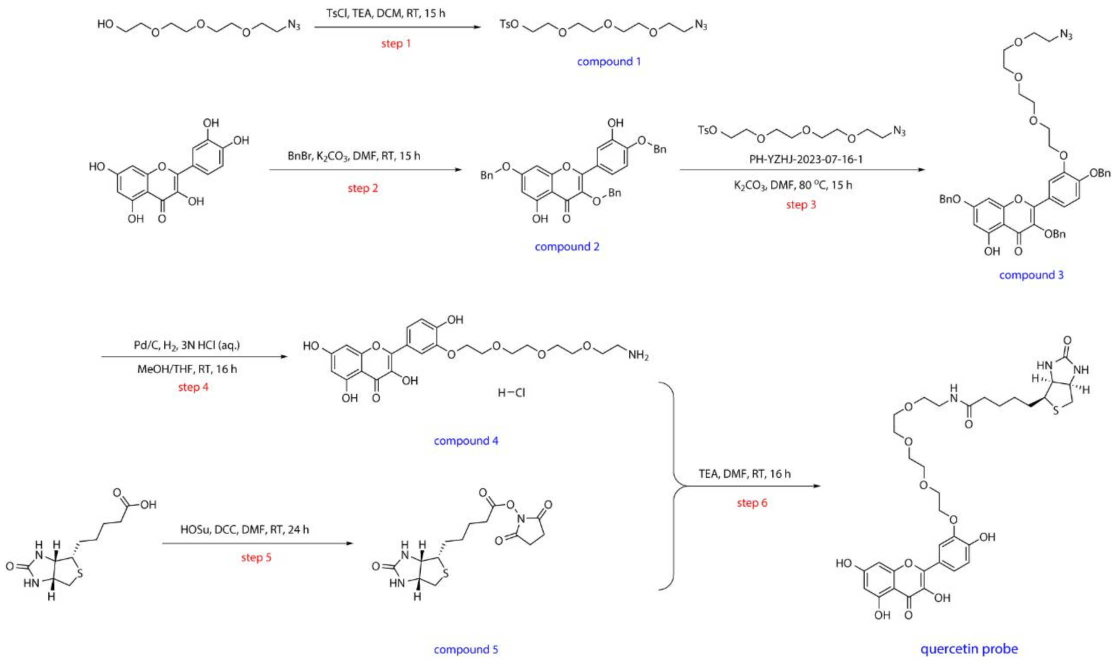

Synthesis route of QR probe

Synthesis of compound 1. To a solution of 2-(2-[2-(2-azidoethoxy)ethoxy]ethoxyethanol (2.00 g, 9.122 mmol, 1 equiv.) and triethylamine (2.77 g, 27.366 mmol, 3.0 equiv.) in dichloromethane (50 mL) was added 4-methylbenzenesulfonyl chloride (2.61 g, 13.683 mmol, 1.5 equiv.). The mixture was stirred at room temperature for 15 h and then concentrated under reduced pressure. The residue was purified by silica gel column chromatography (eluting with ethyl acetate/petroleum ether 1:2) to afford 1-(2-(2-[2-(2-azidoethoxy)ethoxy]ethoxyethoxysulfonyl)-4-methylbenzene (3.00 g, 88.1% yield) as a light yellow oil.

Synthesis of compound 2. To a solution of QR (1.50 g, 4.963 mmol, 1 equiv.) in N,N-Dimethylformamide (80 mL) was added potassium carbonate (2.06 g, 14.889 mmol, 3.0 equiv.). The mixture was cooled to 0 °C and a solution of benzyl bromide (2.55 g, 14.889 mmol, 3.0 equiv.) in N,N-Dimethylformamide (10 mL) was added dropwise under nitrogen atmosphere. After stirring at 0 °C for 2 h, the reaction mixture was allowed to warm to room temperature for 15 h and then concentrated

under reduced pressure. The residue was diluted with water (60 mL) and extracted with ethyl acetate (120 mL). The organic layer was washed with brine (60 mL x 1), then dried over anhydrous sodium sulfate, filtered and concentrated under reduced pressure. The residue was purified by silica gel column chromatography (eluting with ethyl acetate/petroleum ether 1:3) to afford 3,7-bis(benzyloxy)-2-[4-(benzyloxy)-3-hydroxyphenyl]-5-hydroxychromen-4-one (2.10 g, 73.9% yield) as a yellow solid.

Synthesis of compound 3. To a solution of 3,7-bis(benzyloxy)-2-[4-(benzyloxy)-3-hydroxyphenyl]-5-hydroxychromen-4-one (2.10 g, 3.667 mmol, 1 equiv.) in N,N-Dimethylformamide (10.5 mL) was added potassium carbonate (0.66 g, 4.767 mmol, 1.30 equiv.), followed by the addition of 1-(2-(2-[2-(2-azidoethoxy)ethoxy]ethoxyethoxysulfonyl)-4-methylbenzene (1.37 g, 3.667 mmol, 1.00 equiv.) in N,N-Dimethylformamide (3 mL) dropwise. After stirring at 80 °C for 15 h, the reaction mixture was cooled to room temperature and concentrated under reduced pressure. The residue was diluted with water (60 mL) and extracted with ethyl acetate (120 mL). The organic layer was washed with brine (60 mL x 1), then dried over anhydrous sodium sulfate, filtered and concentrated under reduced pressure. The residue was purified by silica gel column chromatography (eluting with ethyl acetate/petroleum ether 1:3) to afford 2-[3-(2-(2-[2-(2-azidoethoxy)ethoxy]ethoxyethoxy)-4-(benzyloxy)phenyl]-3,7-bis(benzyloxy)-5-hydroxychromen-4-one (1.44 g, 50.7% yield) as a light-yellow solid.

Synthesis of compound 4. To a solution of 2-[3-(2-(2-[2-(2-azidoethoxy)ethoxy]ethoxyethoxy)-4-(benzyloxy)phenyl]-3,7-bis(benzyloxy)-5-hydroxychromen-4-one (800 mg, 1.034 mmol, 1 equiv.) in methanol (10 mL) and Tetrahydrofuran (10 mL) were added 10% Pd/C (160 mg, 0.150 mmol, 0.15 equiv., 10%) and 3N hydrochloric acid (0.5 mL, 1.500 mmol, 1.45 equiv.). The mixture was purged with hydrogen gas and stirred at room temperature for 16 h under hydrogen atmosphere (2 atm). The reaction mixture was filtered through a celite pad,

and the filtrate was concentrated under reduced pressure. The residue was purified by reversed phase column chromatography (eluting with 40% methanol in water) and then concentrated under reduced pressure to afford 2-[3-(2-(2-[2-(2-aminoethoxy)ethoxy]ethoxyethoxy)-4-hydroxyphenyl]-3,5,7-trihydroxychromen-4-one hydrochloride (300 mg, 56.5% yield) as a yellow solid.

Synthesis of compound 5. To a solution of biotin (0.50 g, 2.047 mmol, 1 equiv.) and N-hydroxysuccinimide (0.24 g, 2.047 mmol, 1.0 equiv.) in N,N-Dimethylformamide (40 mL) was added DCC (0.55 g, 2.661 mmol, 1.3 equiv.). The mixture was stirred at room temperature for 24 h. The solid precipitate was removed by filtration, and the filtrate was concentrated under reduced pressure. The residue was triturated with methanol (20 mL) twice and filtered. The solid was collected and then dried under reduced pressure to afford 2,5-dioxopyrrolidin-1-yl 5-[(3aS,4S,6aR)-2-oxo-hexahydrothieno[3,4-d]imidazol-4-yl]pentanoate (0.55 g, 61.4% yield, 78% purity) as a white solid.

Synthesis of QR probe. To a solution of 2-[3-(2-(2-[2-(2-aminoethoxy)ethoxy]ethoxyethoxy)-4-hydroxyphenyl]-3,5,7-trihydroxychromen-4-one hydrochloride (200 mg, 0.389 mmol, 1 equiv.) in N,N-Dimethylformamide (6 mL) were added 2,5-dioxopyrrolidin-1-yl 5-[(3aS,4S,6aR)-2-oxo-hexahydrothieno[3,4-d]imidazol-4-yl]pentanoate (204 mg, 0.466 mmol, 1.20 equiv., 78% purity) and triethylamine (80 mg, 0.791 mmol, 2.03 equiv.). After stirring at room temperature for 16 h, the reaction mixutre was concentrated under reduced pressure. The residue was purified by reversed phase column chromatography (eluting with 60% methanol in water) to afford 140 mg of 5-[(3aS,4S,6aR)-2-oxo-hexahydrothieno[3,4-d]imidazol-4-yl]-N-(2-[2-(2-(2-[2-hydroxy-5-(3,5,7-trihydroxy-4-oxochromen-2-yl)phenoxy]ethoxyethoxy)ethoxy]ethylpentanamide (140 mg, 50.8% yield) as a yellow solid.
