# Supplementary material for: Quercetin, a flavonoid, suppresses viral proliferation by interfering with the ubiquitin transfer from E1 to E2 enzymes
Source: PLoS Pathog. 2026 Jul 20;22(7):e1014425. doi: 10.1371/journal.ppat.1014425 (PMC13399506; doi:10.1371/journal.ppat.1014425)

Figure 1

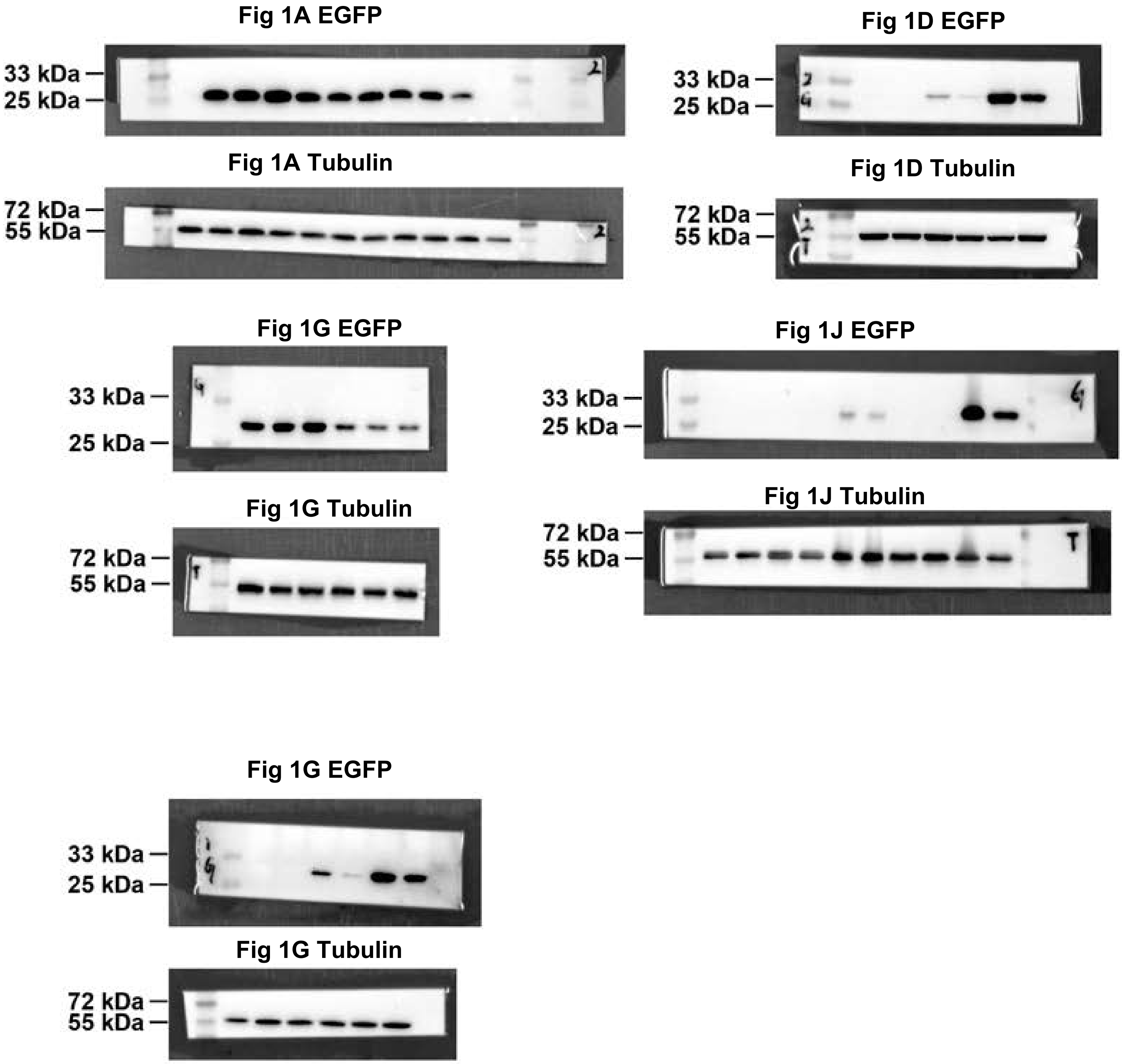
Fig 1A EGFP
33 kDa
25 kDa
Fig 1A Tubulin
72 kDa
55 kDa
Fig 1D EGFP
33 kDa
25 kDa
Fig 1D Tubulin
72 kDa
55 kDa
Fig 1G EGFP
33 kDa
25 kDa
Fig 1G Tubulin
72 kDa
55 kDa
Fig 1J EGFP
33 kDa
25 kDa
Fig 1J Tubulin
72 kDa
55 kDa
Fig 1G EGFP
33 kDa
25 kDa
Fig 1G Tubulin
72 kDa
55 kDa

Figure 2

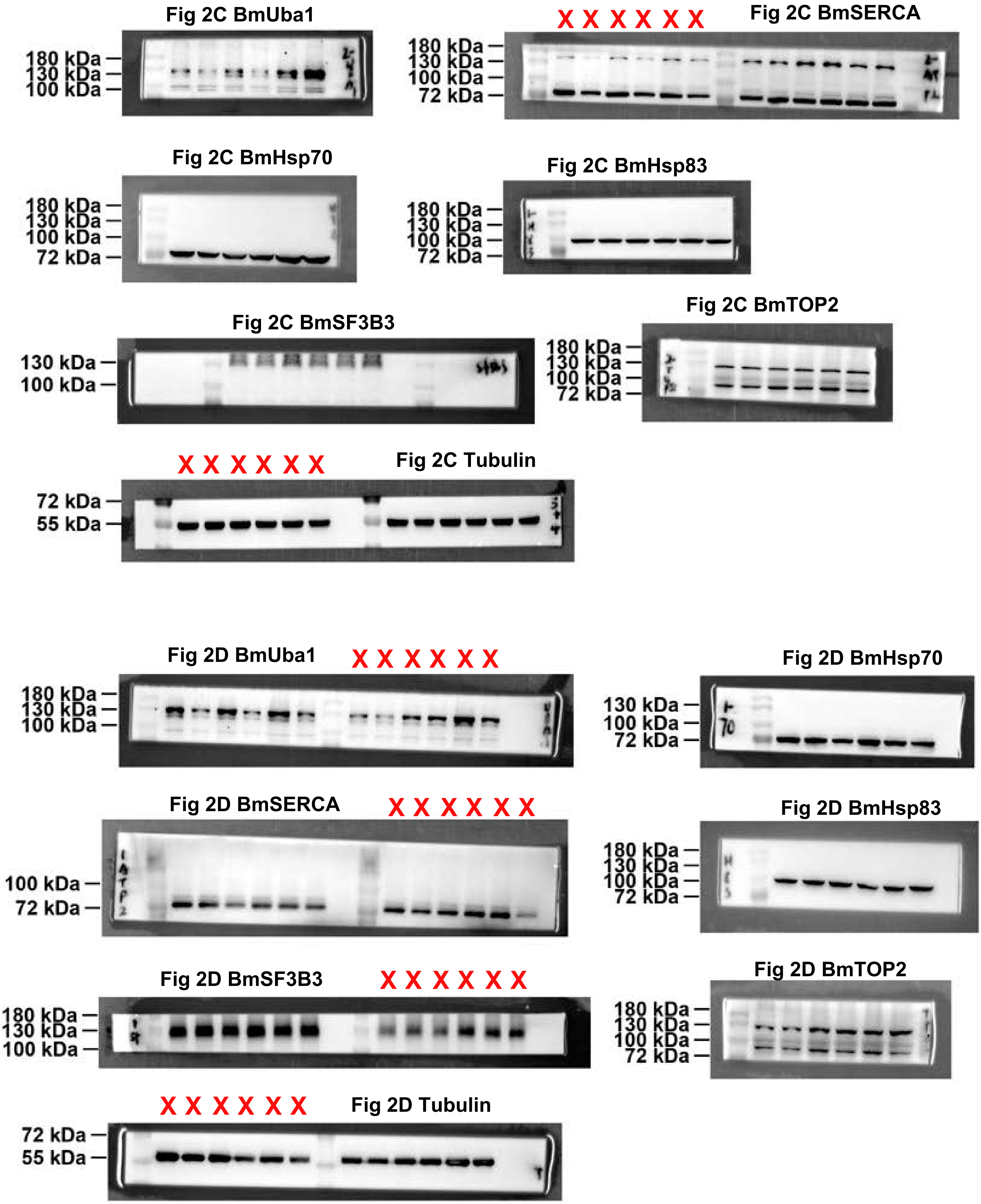
Fig 2C BmUba1
180 kDa
130 kDa
100 kDa
X X X X X X
Fig 2C BmSERCA
180 kDa
130 kDa
100 kDa
72 kDa
Fig 2C BmHsp70
180 kDa
130 kDa
100 kDa
72 kDa
Fig 2C BmHsp83
180 kDa
130 kDa
100 kDa
72 kDa
Fig 2C BmSF3B3
130 kDa
100 kDa
Fig 2C BmTOP2
180 kDa
130 kDa
100 kDa
72 kDa
X X X X X X
Fig 2C Tubulin
72 kDa
55 kDa
Fig 2D BmUba1
X X X X X X
180 kDa
130 kDa
100 kDa
Fig 2D BmHsp70
130 kDa
100 kDa
72 kDa
Fig 2D BmSERCA
X X X X X X
100 kDa
72 kDa
Fig 2D BmHsp83
180 kDa
130 kDa
100 kDa
72 kDa
Fig 2D BmSF3B3
X X X X X X
180 kDa
130 kDa
100 kDa
Fig 2D BmTOP2
180 kDa
130 kDa
100 kDa
72 kDa
X X X X X X
Fig 2D Tubulin
72 kDa
55 kDa

Figure 3

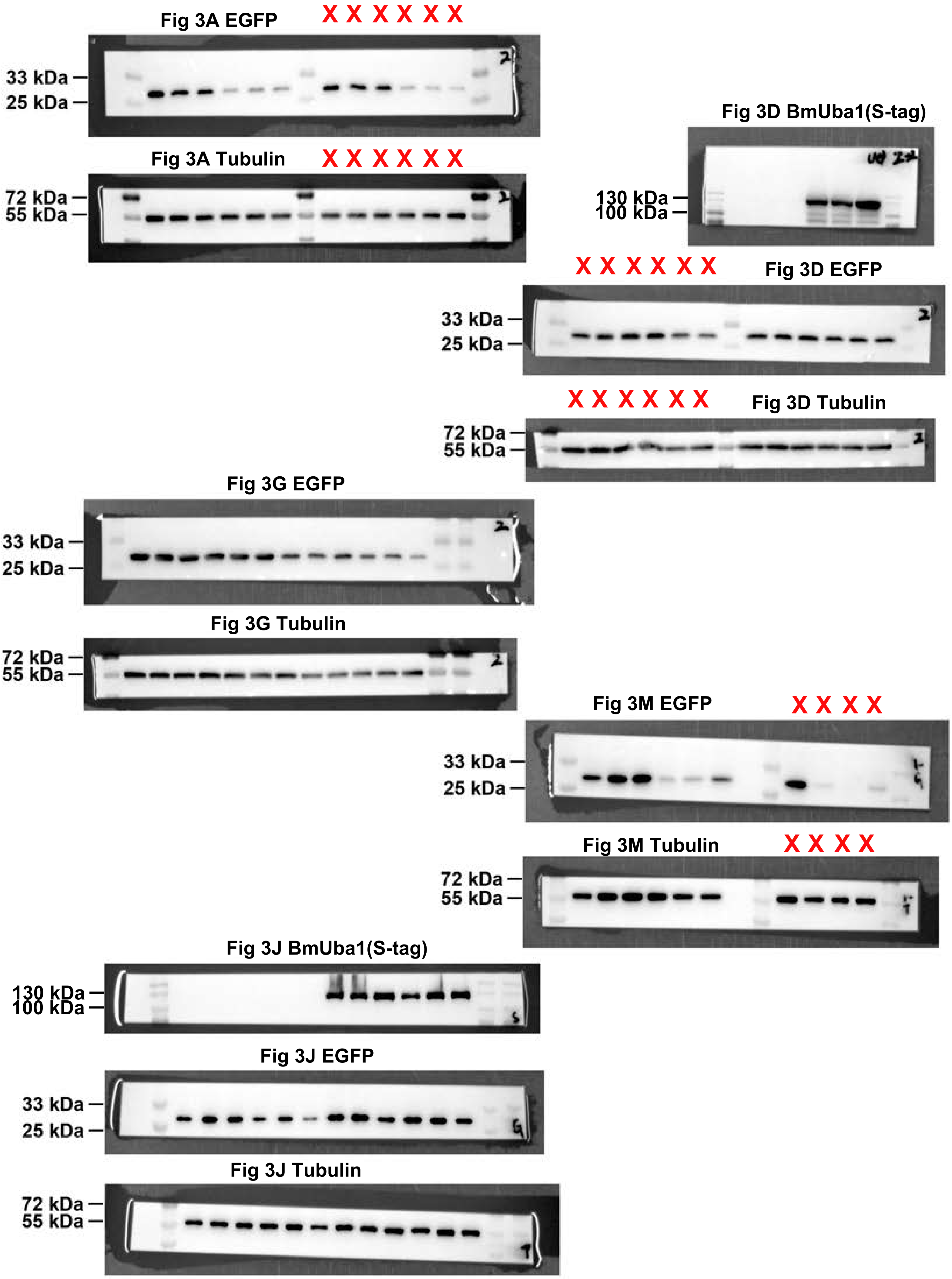
Fig 3A EGFP
X X X X X X
33 kDa
25 kDa
Fig 3A Tubulin
X X X X X X
72 kDa
55 kDa
Fig 3D BmUba1(S-tag)
130 kDa
100 kDa
X X X X X X
Fig 3D EGFP
33 kDa
25 kDa
X X X X X X
Fig 3D Tubulin
72 kDa
55 kDa
Fig 3G EGFP
33 kDa
25 kDa
Fig 3G Tubulin
72 kDa
55 kDa
Fig 3M EGFP
X X X X
33 kDa
25 kDa
Fig 3M Tubulin
X X X X
72 kDa
55 kDa
Fig 3J BmUba1(S-tag)
130 kDa
100 kDa
Fig 3J EGFP
33 kDa
25 kDa
Fig 3J Tubulin
72 kDa
55 kDa

Figure 4

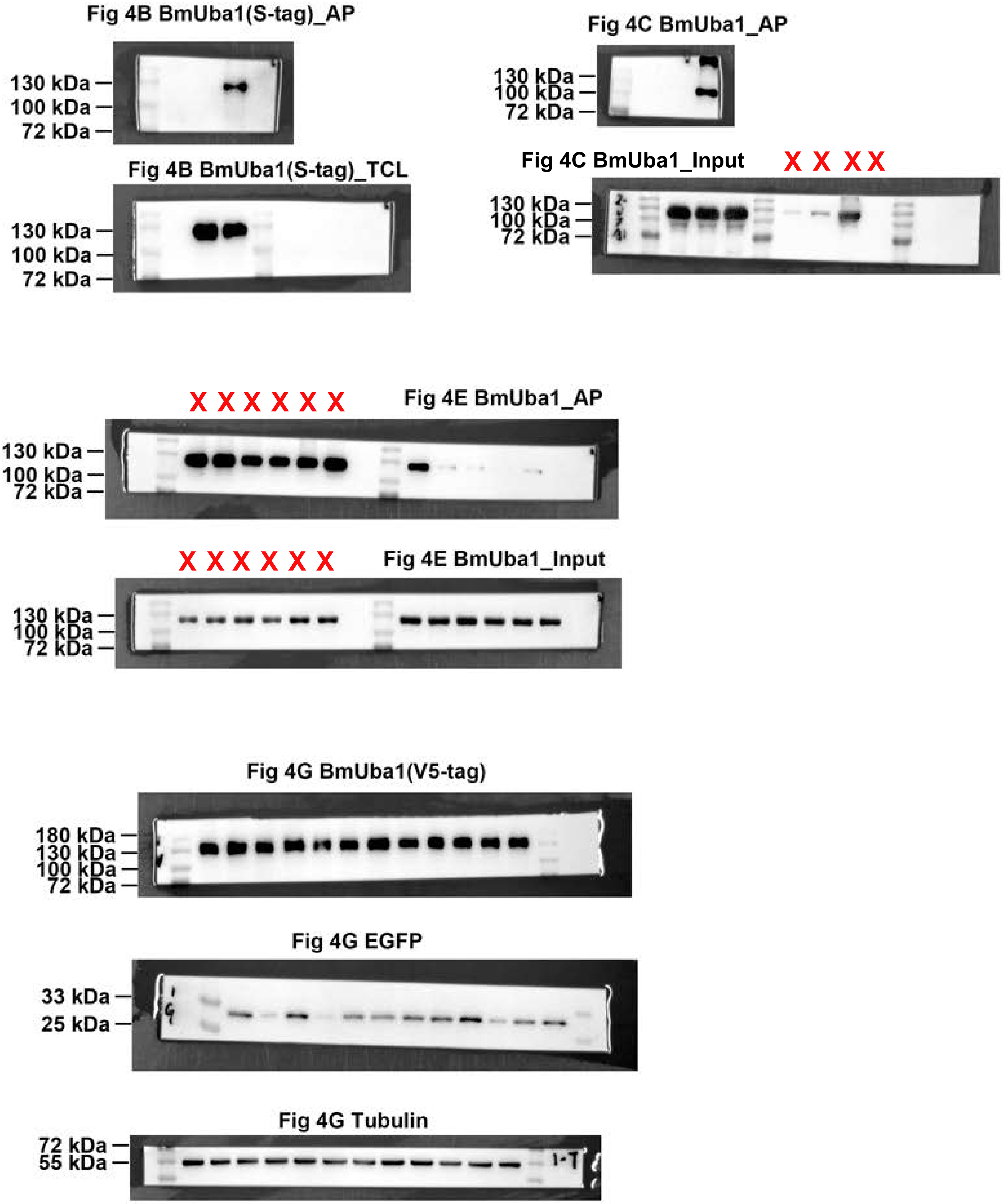
Fig 4B BmUba1(S-tag)_AP
130 kDa
100 kDa
72 kDa
Fig 4B BmUba1(S-tag)_TCL
130 kDa
100 kDa
72 kDa
Fig 4C BmUba1_AP
130 kDa
100 kDa
72 kDa
Fig 4C BmUba1_Input
130 kDa
100 kDa
72 kDa
Fig 4E BmUba1_AP
130 kDa
100 kDa
72 kDa
Fig 4E BmUba1_Input
130 kDa
100 kDa
72 kDa
Fig 4G BmUba1(V5-tag)
180 kDa
130 kDa
100 kDa
72 kDa
Fig 4G EGFP
33 kDa
25 kDa
Fig 4G Tubulin
72 kDa
55 kDa

Figure 5_1

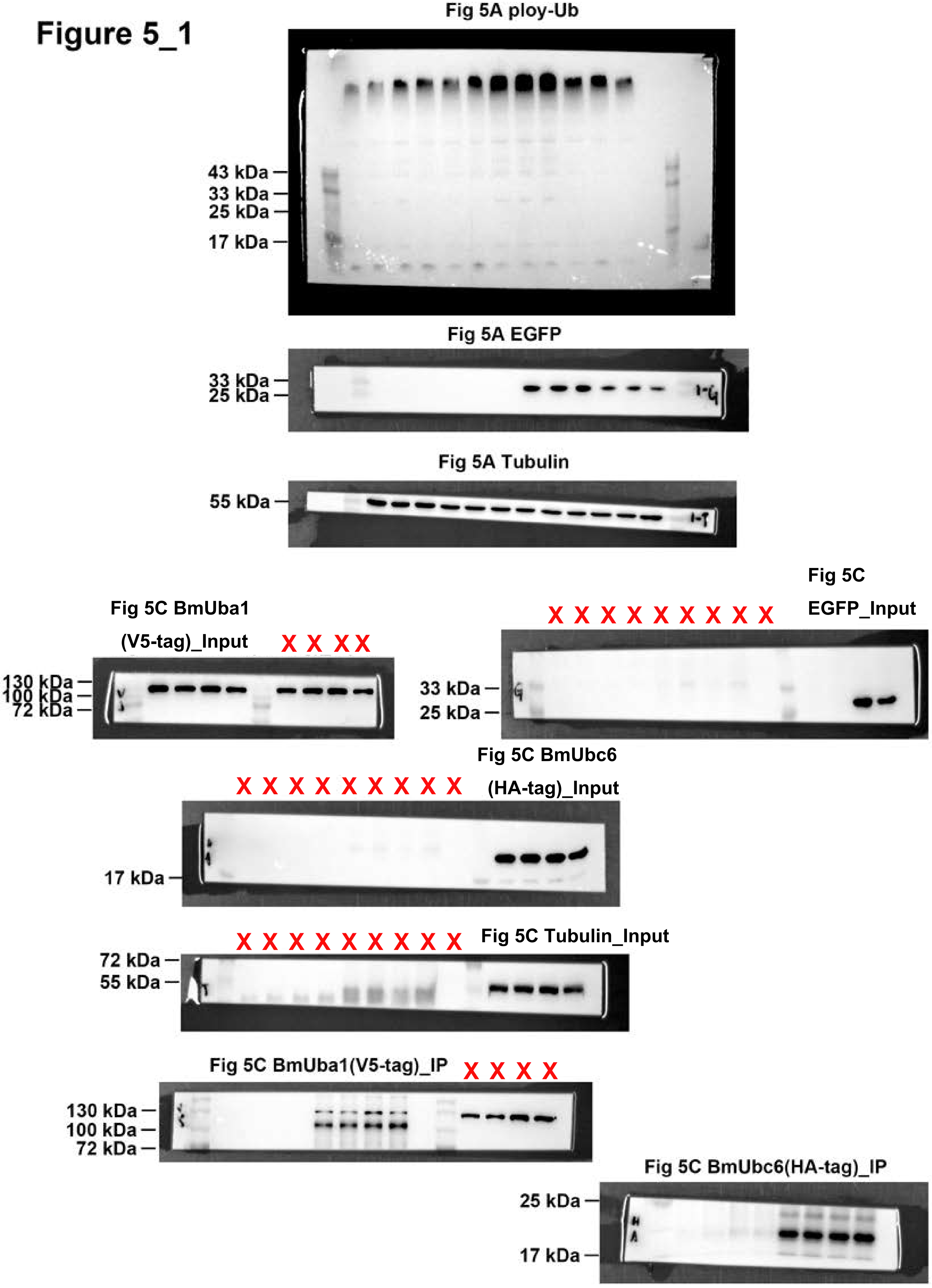
Fig 5A ploy-Ub
43 kDa
33 kDa
25 kDa
17 kDa
Fig 5A EGFP
33 kDa
25 kDa
Fig 5A Tubulin
55 kDa
Fig 5C BmUba1
(V5-tag)_Input
130 kDa
100 kDa
72 kDa
Fig 5C
EGFP_Input
33 kDa
25 kDa
Fig 5C BmUbc6
(HA-tag)_Input
17 kDa
Fig 5C Tubulin_Input
72 kDa
55 kDa
Fig 5C BmUba1(V5-tag)_IP
130 kDa
100 kDa
72 kDa
Fig 5C BmUbc6(HA-tag)_IP
25 kDa
17 kDa

Figure 5_2

Fig 5D

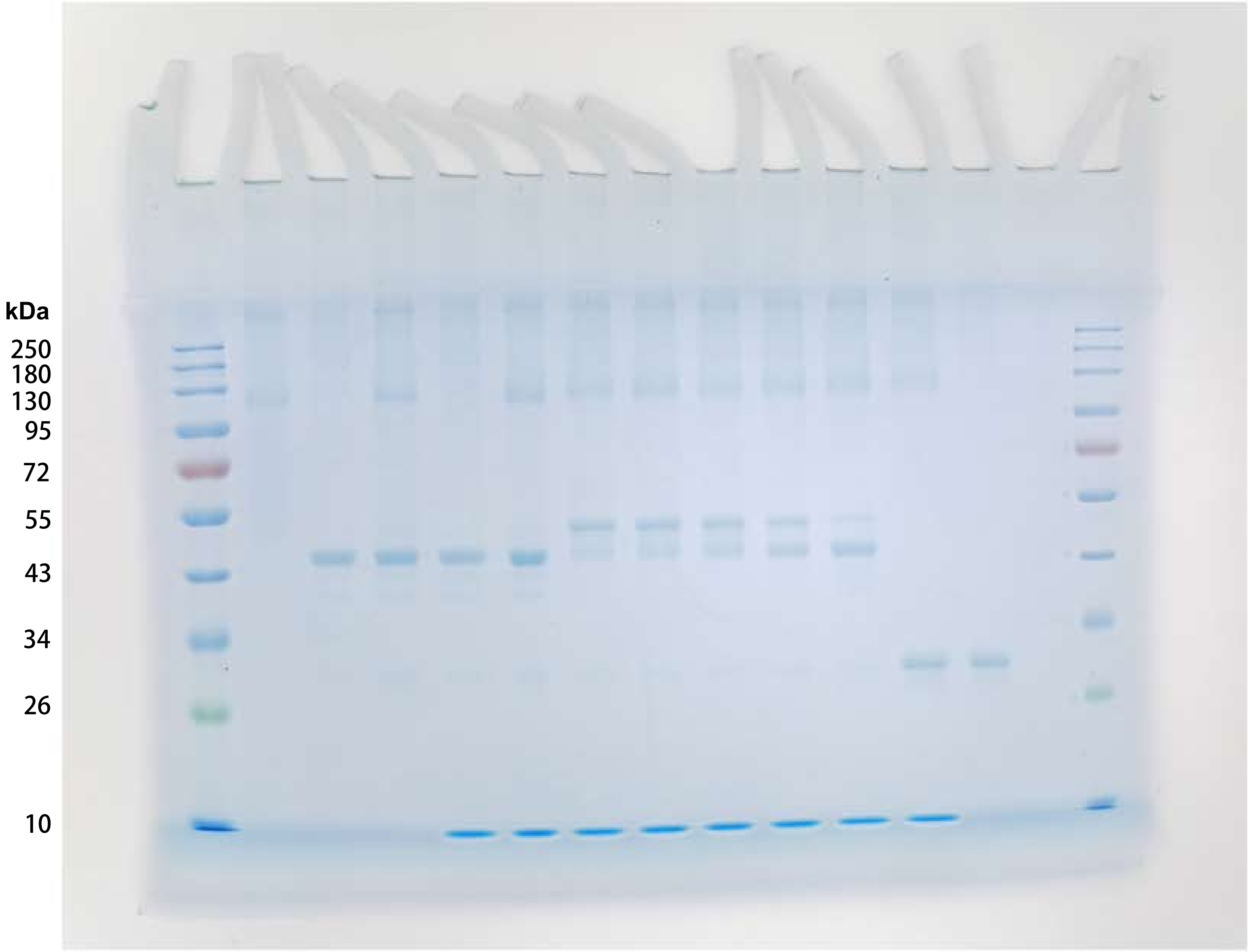

Fig 5E EGFP

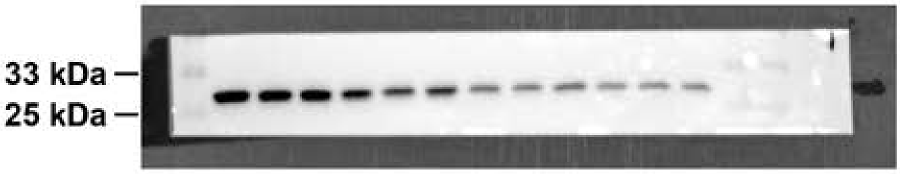

Fig 5E Tubulin

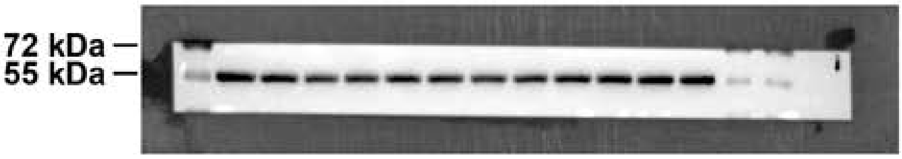

Figure 5_3

Fig 5H BmUbc6(HA-tag)

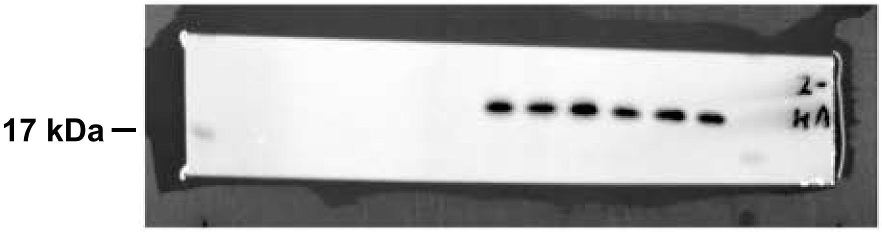

Fig 5H EGFP

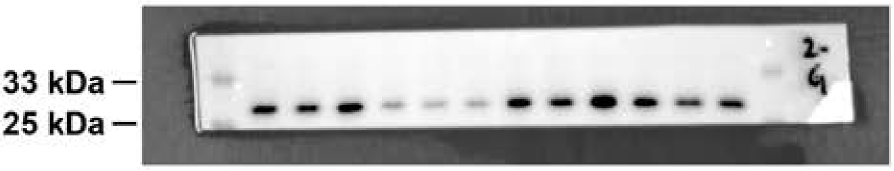

Fig 5H Tubulin

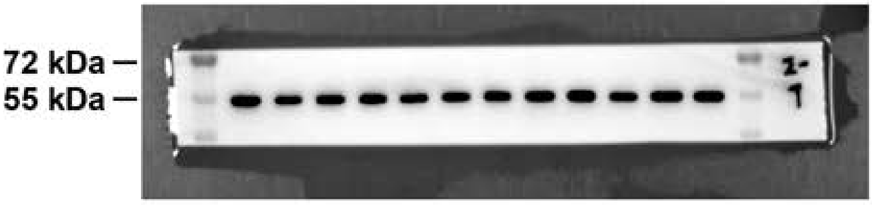

Fig 5K poly-Ub

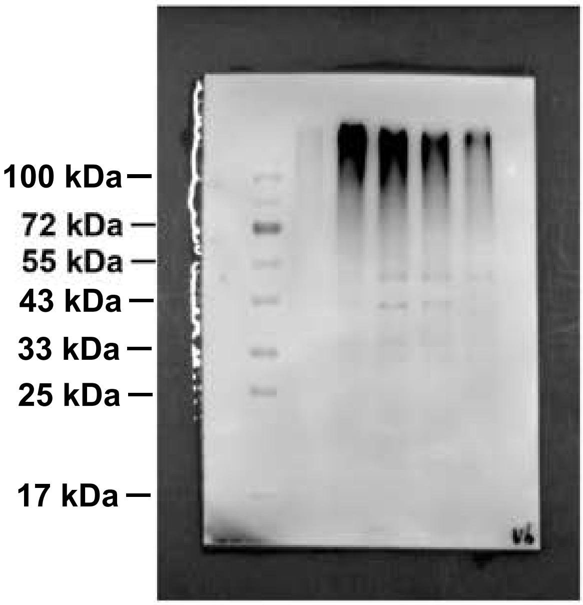

Fig 5K BmAurka-b (FLAG-tag)_IP

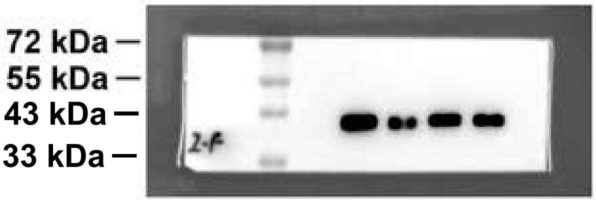

Fig 5K Tubulin

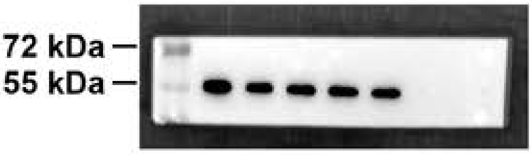

Fig 5K BmAurka-b

(FLAG-tag)_Input

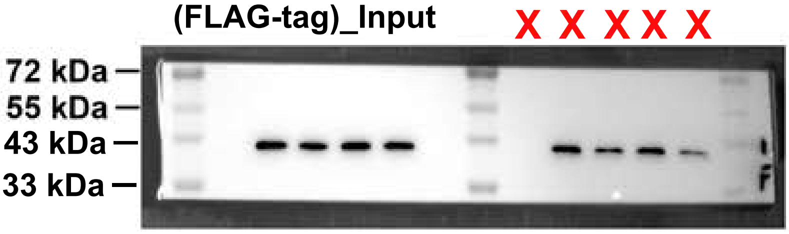

Figure 6

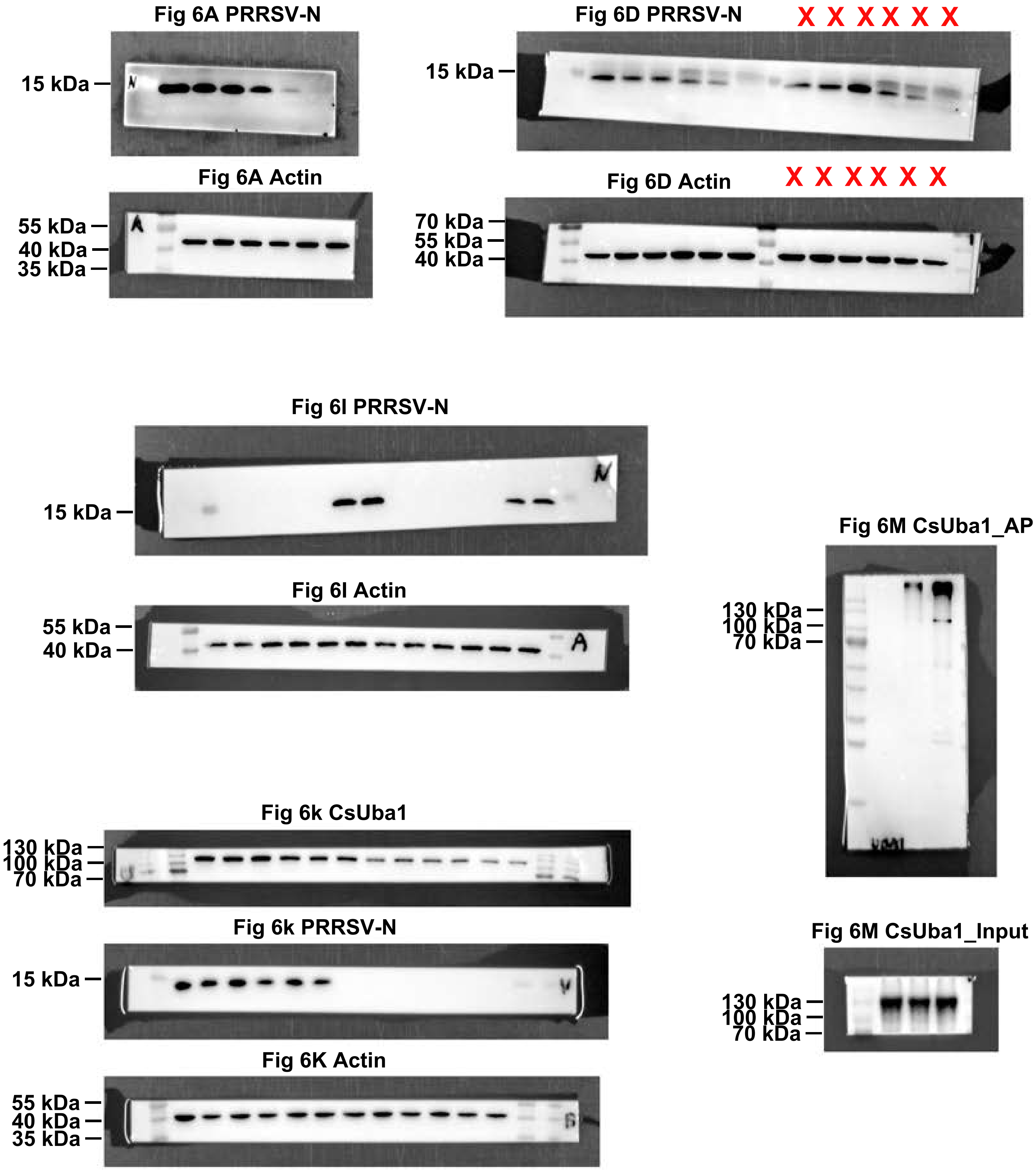
Fig 6A PRRSV-N
15 kDa
Fig 6A Actin
55 kDa
40 kDa
35 kDa
Fig 6D PRRSV-N
X X X X X X
15 kDa
Fig 6D Actin
X X X X X X
70 kDa
55 kDa
40 kDa
Fig 6I PRRSV-N
15 kDa
Fig 6I Actin
55 kDa
40 kDa
Fig 6M CsUba1_AP
130 kDa
100 kDa
70 kDa
Fig 6k CsUba1
130 kDa
100 kDa
70 kDa
Fig 6k PRRSV-N
15 kDa
Fig 6K Actin
55 kDa
40 kDa
35 kDa
Fig 6M CsUba1_Input
130 kDa
100 kDa
70 kDa

Figure S2

Fig S2E BmUba1

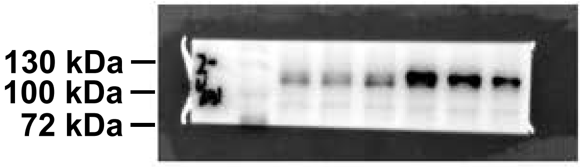

Fig S2E BmUba1 (V5-tag)

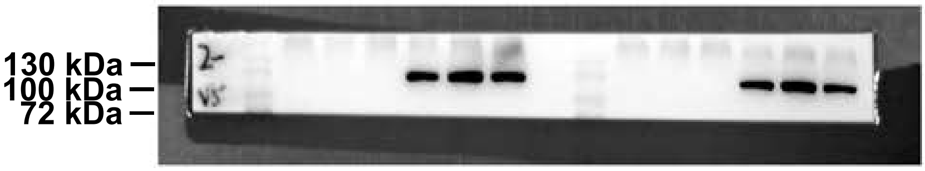

Fig S2E Tubulin

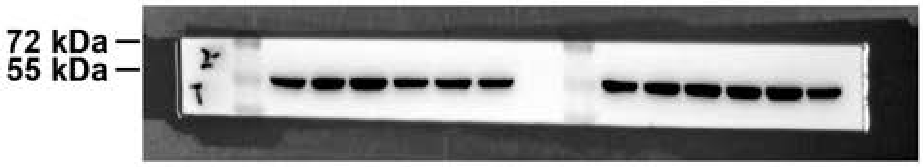

Figure S3

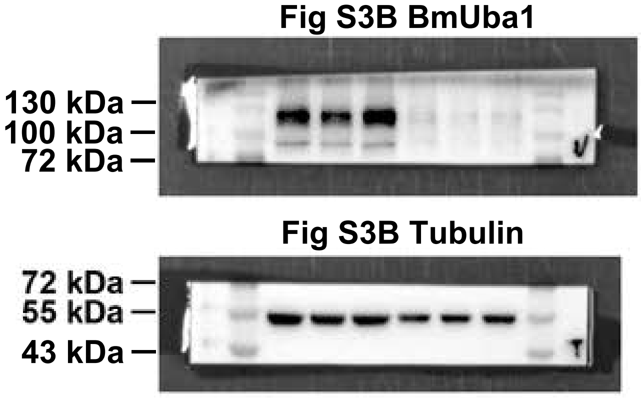
Fig S3B BmUba1
130 kDa
100 kDa
72 kDa
Fig S3B Tubulin
72 kDa
55 kDa
43 kDa

Figure S6

Fig S6B

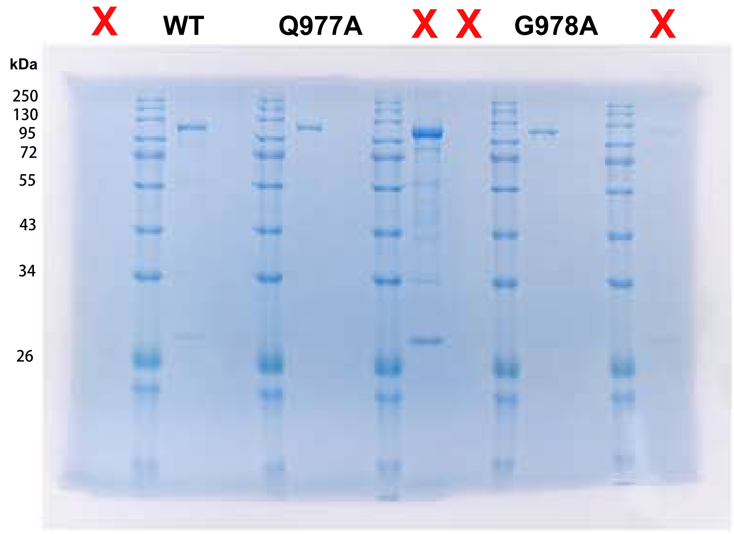

Fig S6B

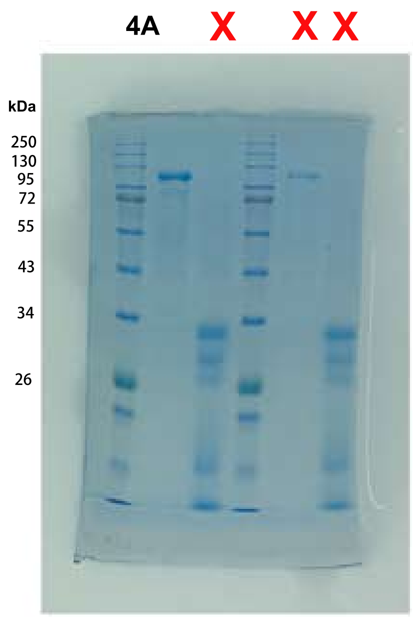

Fig S6B

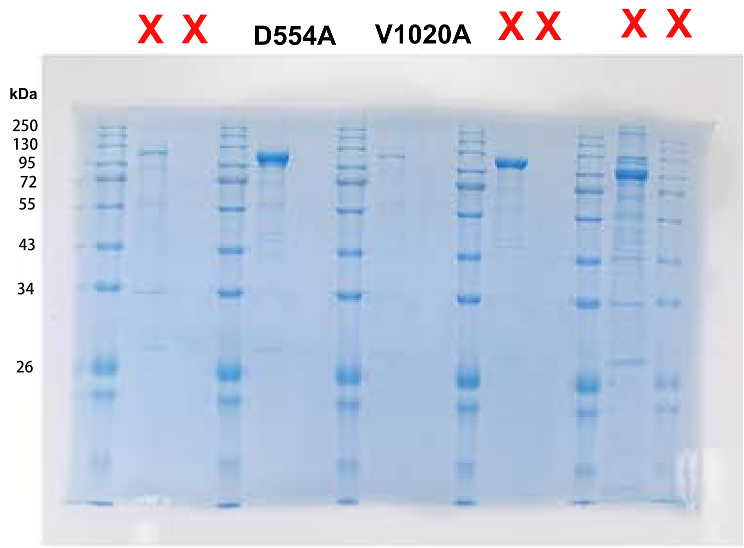

Fig S6D BmUba1 (V5-tag)

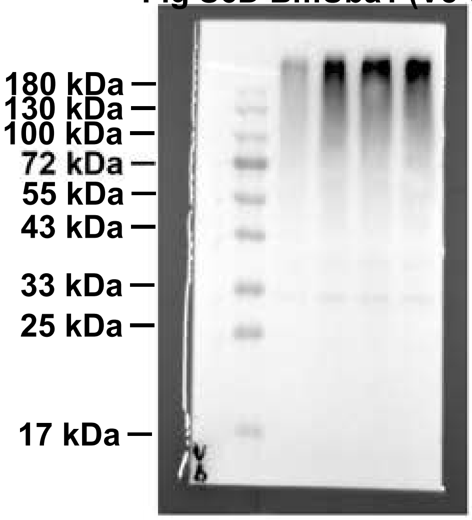

Fig S6D BmUba1 (V5-tag)

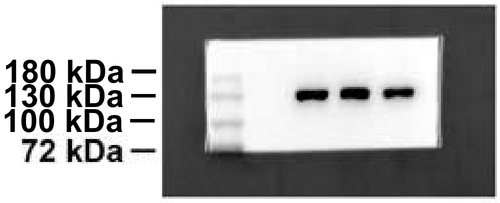

Fig S6D Tubulin

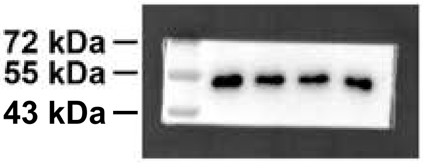

Figure S7

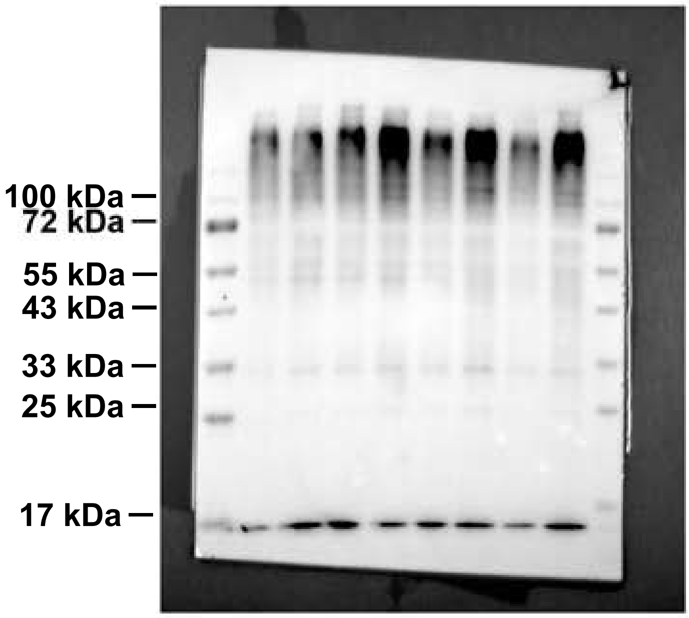

Fig S7A poly-Ub

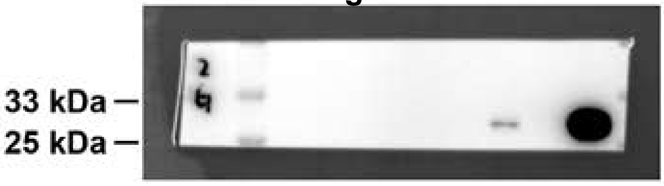

Fig S7A EGFP

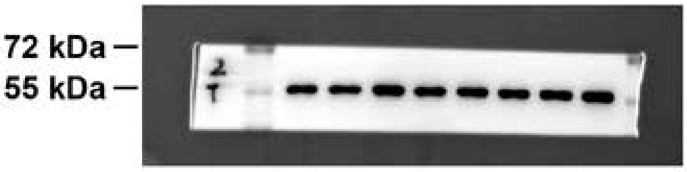

Fig S7A Tubulin

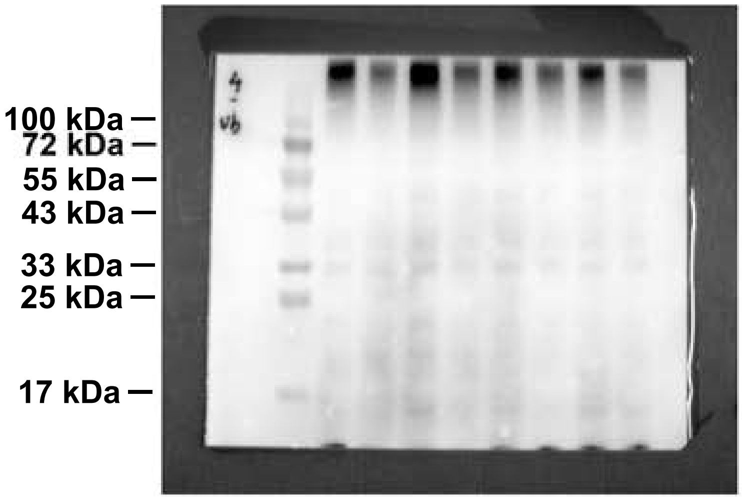

Fig S7B poly-Ub

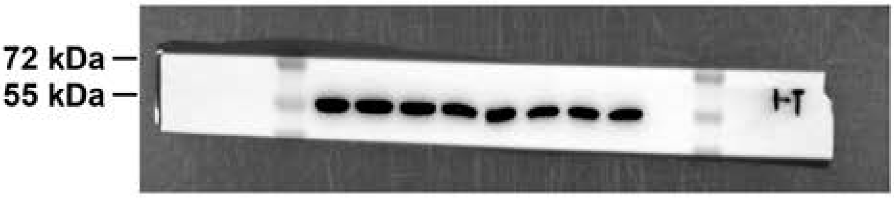

Fig S7B Tubulin

Figure S8_1

Fig S8A BmUba1 (V5-tag)_Input

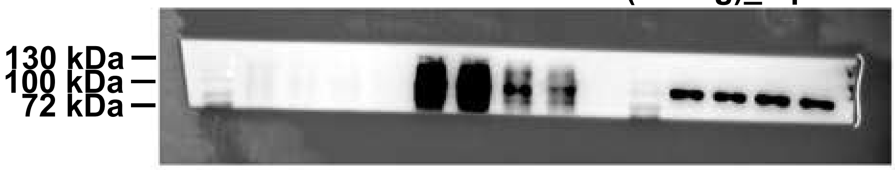

Fig S8A EGFP_Input

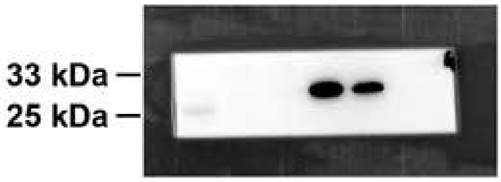

Fig S8A BmUbc13(FLAG-tag)_Input

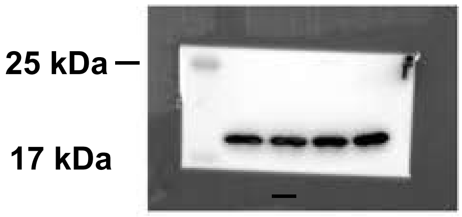

Fig S8A Tubulin_Input

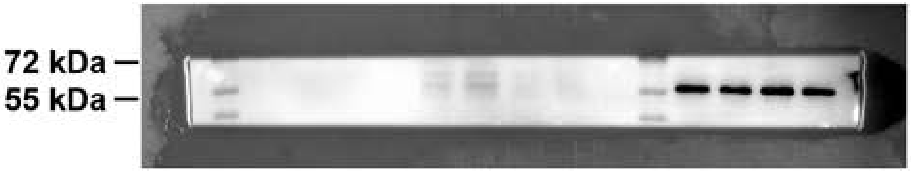

Fig S8A BmUba1(V5-tag)_IP

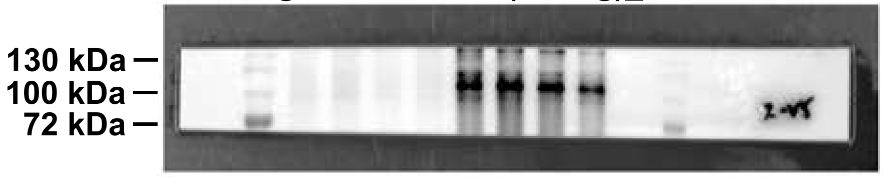

Fig S8A BmUbc13(FLAG-tag)_IP

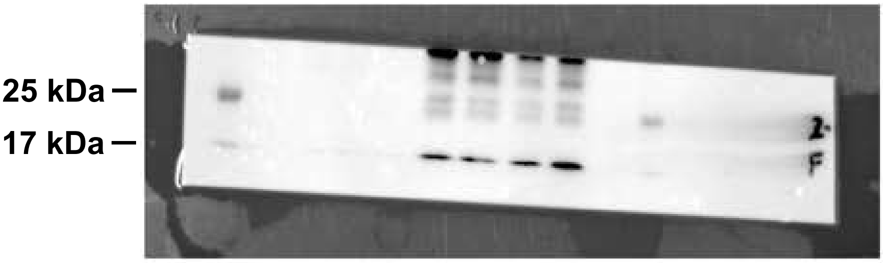

Fig S8B (BmUbc13_GST)

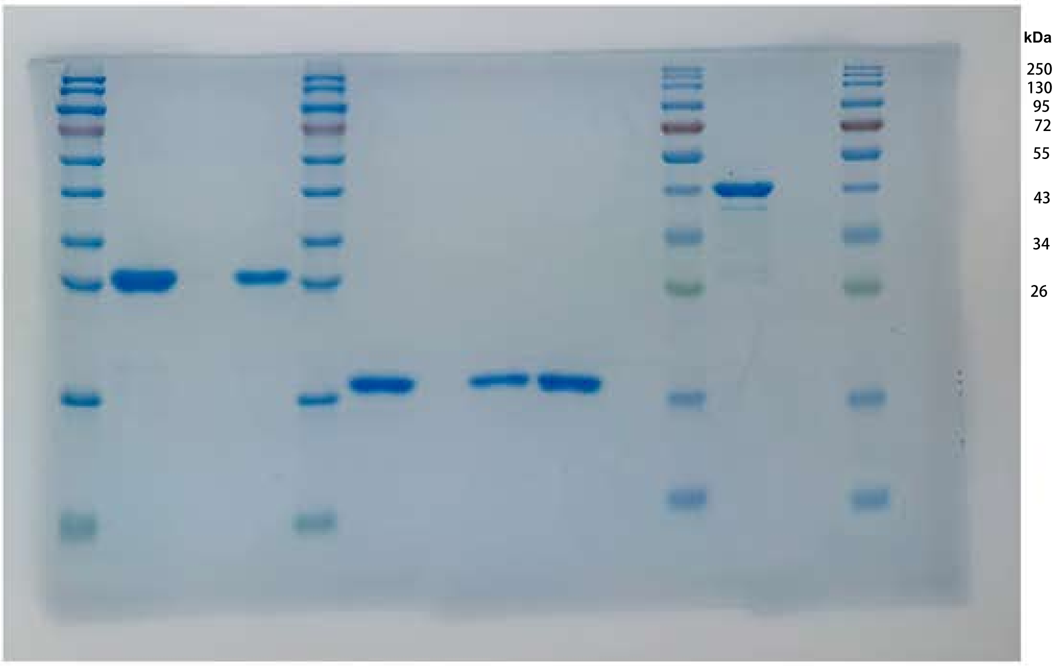

Figure S8_2

Fig S8B

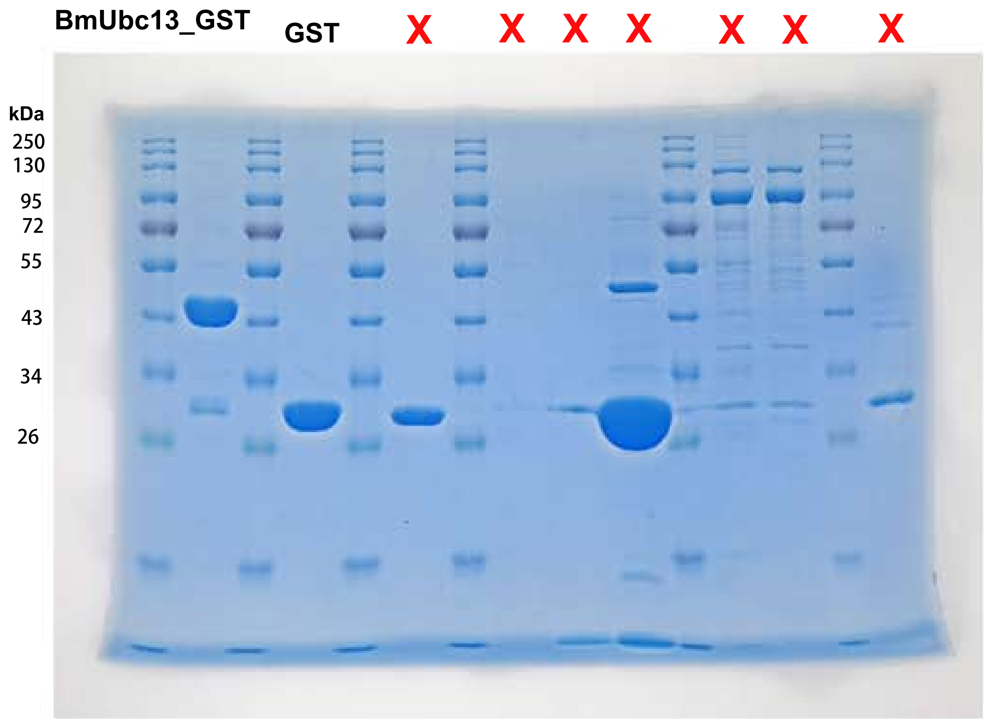

Fig S8B

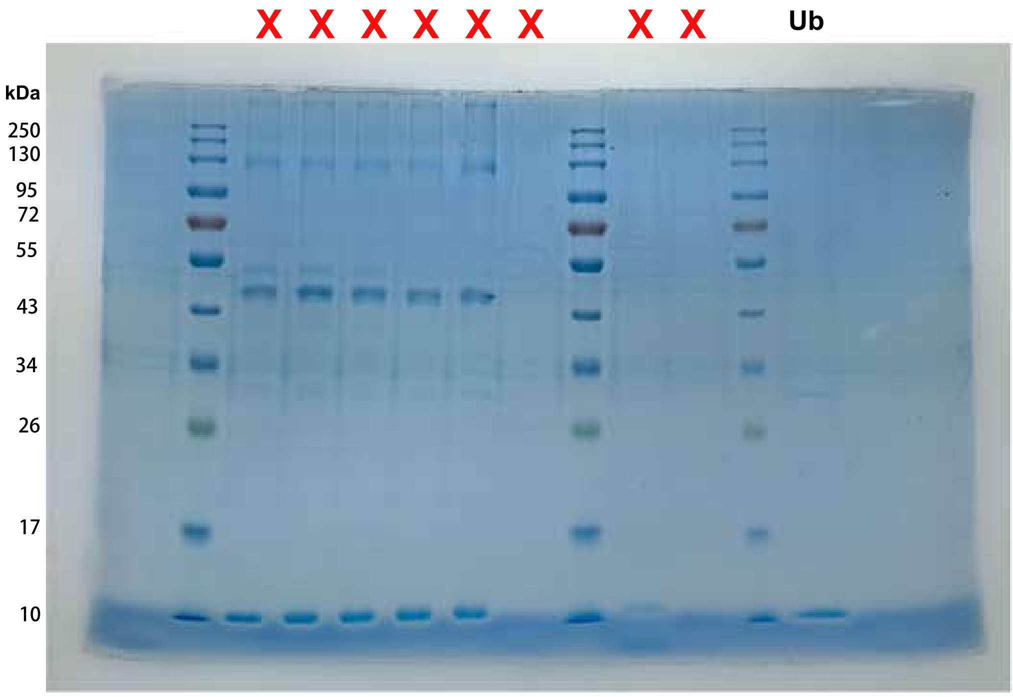

Fig S8C

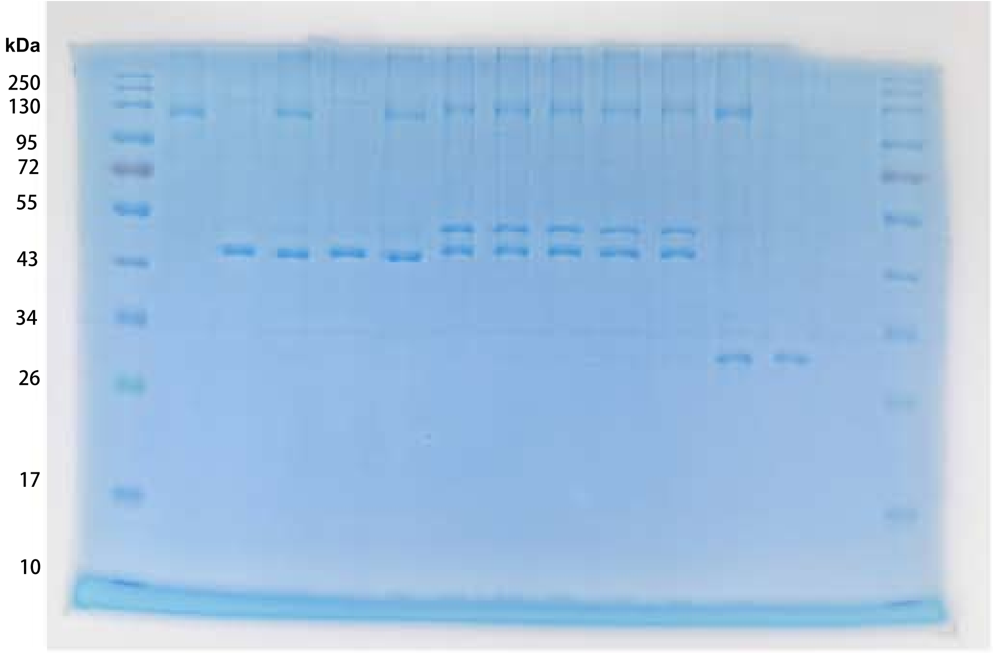

Figure S8_3

Fig S8D

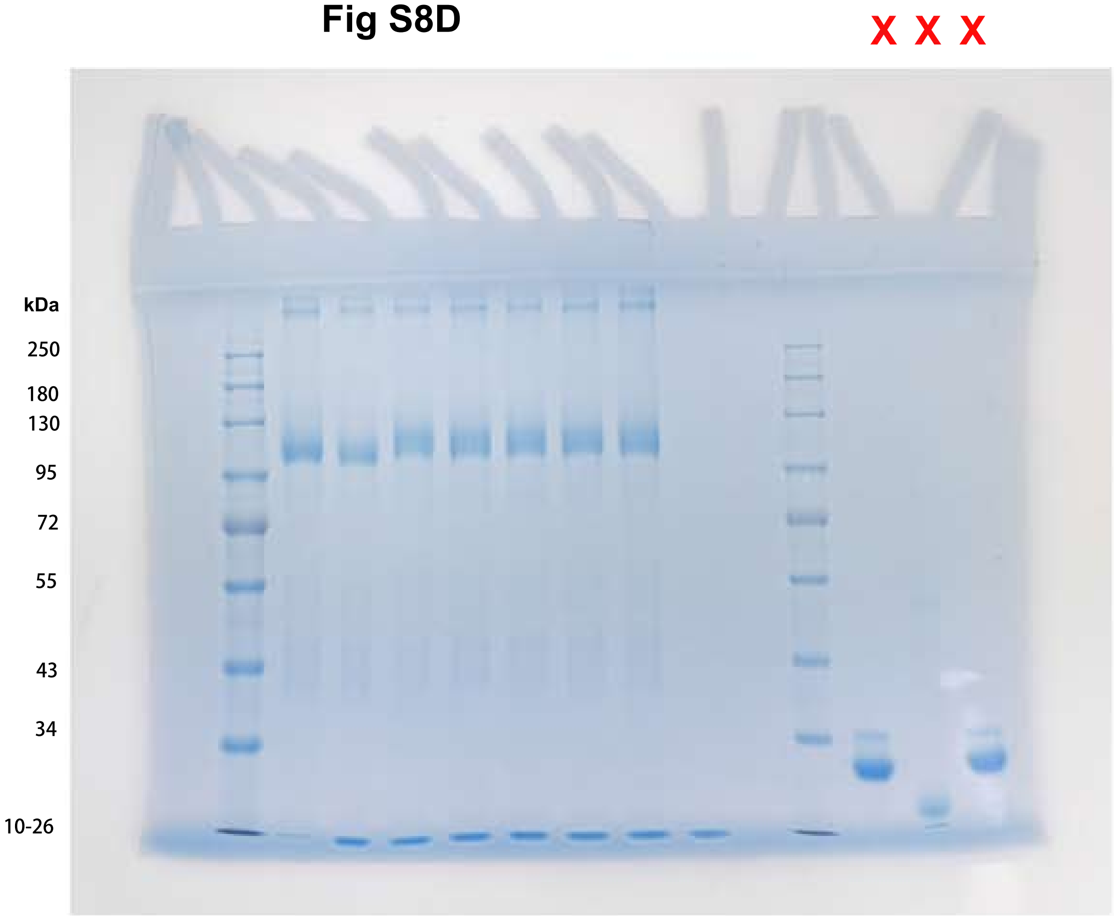

Figure S9

Fig S9B EGFP

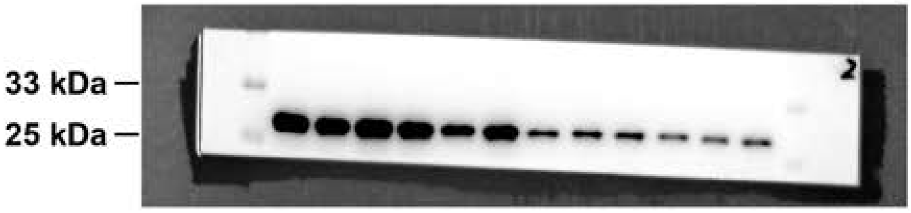

Fig S9B Tubulin

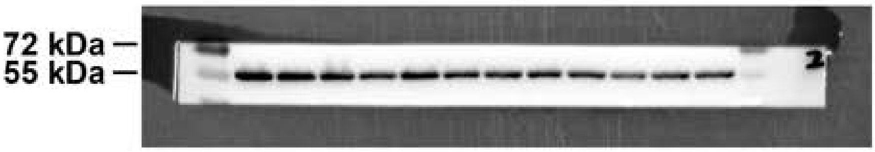

Fig S9G BmUbc13(FLAG-tag)

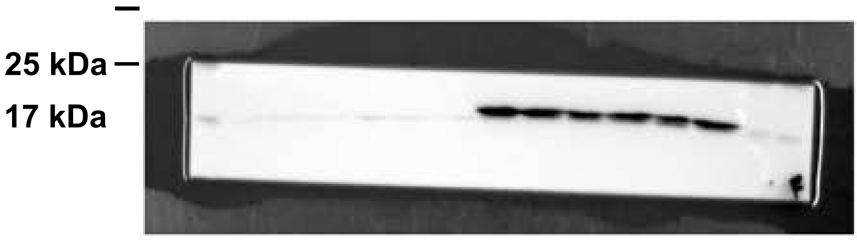

Fig S9G EGFP

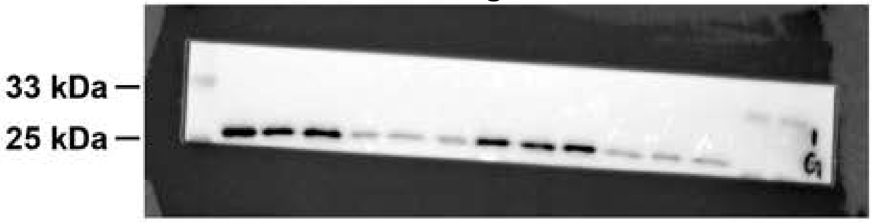

Fig S9G Tubulin

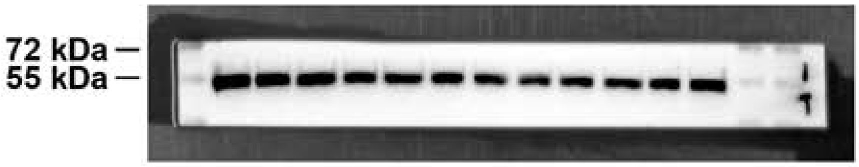

Fig S9J BmUbc6

(HA-tag)_Input

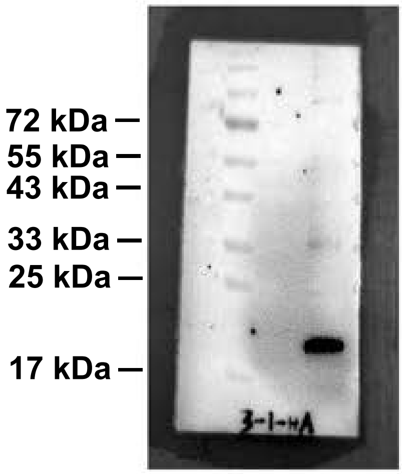

X X X

Fig S9J BmAurka-b

(FLAG-tag)_Input

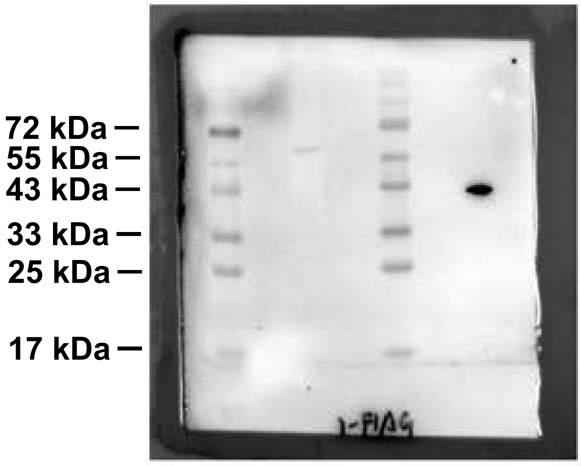

Fig S9J BmUbc6

(HA-tag)_IP

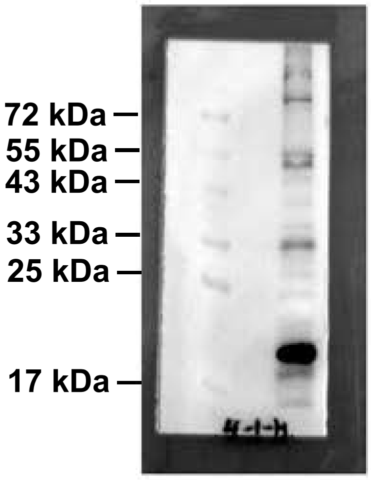

Fig S9J BmAurka-b

(HA-tag)_IP

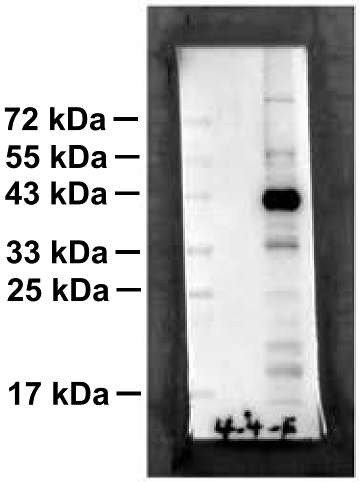

Figure S10

Fig S10G PRRSV-N

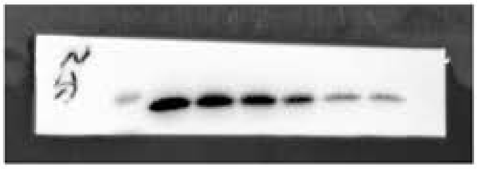

Fig S10G Actin

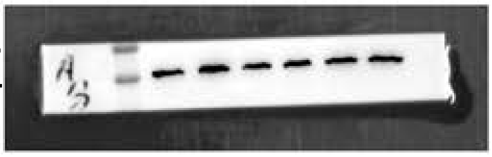

Fig S10K CsUba1

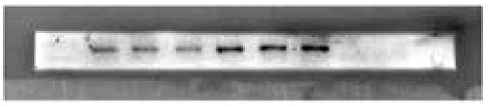

Fig S10K Actin

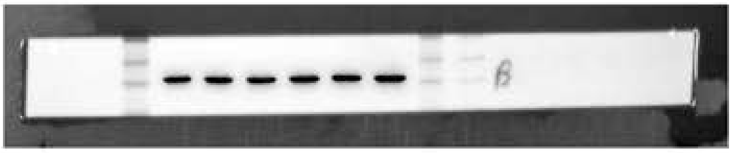

Fig S10L CsUba1

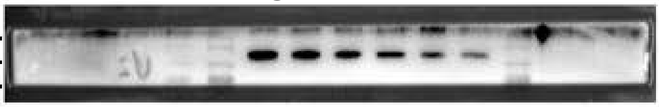

Fig S10L Actin

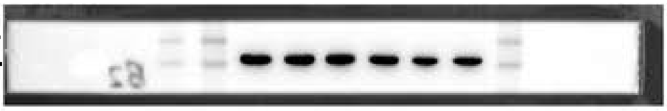

Fig S10N

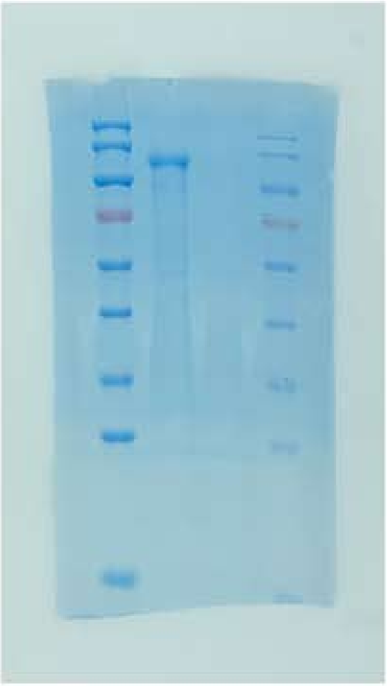

Fig S10O poly-Ub

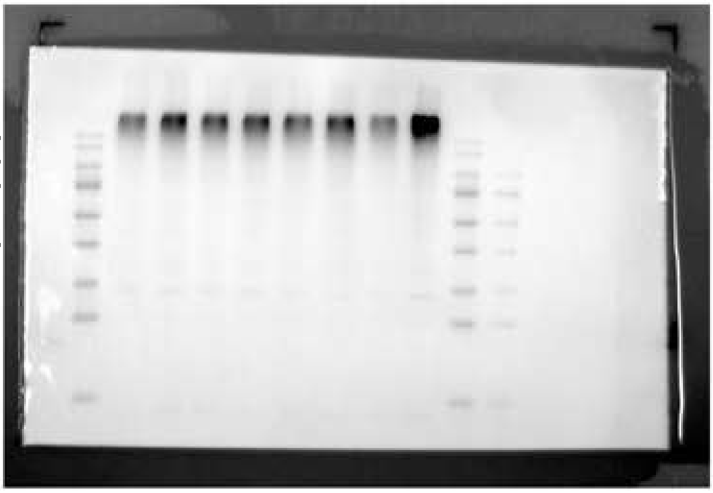

Fig S10O PRRSV-N

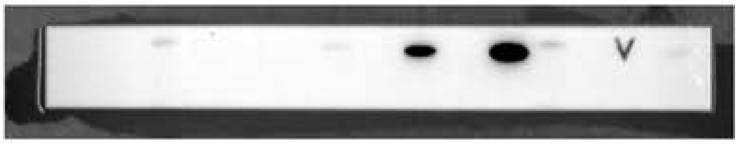

Fig S10O Actin

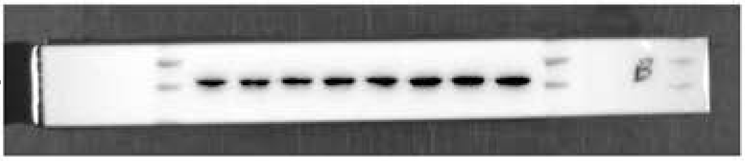

Supplement: S1 Raw Images — (PDF) [file ppat.1014425.s025.pdf]
